# Molecular Signatures of Membrane Protein Complexes Underlying Muscular Dystrophy* [Fn FN2]

**DOI:** 10.1074/mcp.M116.059188

**Published:** 2016-04-20

**Authors:** Rolf Turk, Jordy J. Hsiao, Melinda M. Smits, Brandon H. Ng, Tyler C. Pospisil, Kayla S. Jones, Kevin P. Campbell, Michael E. Wright

**Affiliations:** From the ‡Howard Hughes Medical Institute,; §Senator Paul D. Wellstone Muscular Dystrophy Cooperative Research Center,; ¶Department of Molecular Physiology and Biophysics,; ‖Department of Neurology,; **Department of Internal Medicine, Roy J. and Lucille A. Carver College of Medicine, The University of Iowa, Iowa City, Iowa

## Abstract

Mutations in genes encoding components of the sarcolemmal dystrophin-glycoprotein complex (DGC) are responsible for a large number of muscular dystrophies. As such, molecular dissection of the DGC is expected to both reveal pathological mechanisms, and provides a biological framework for validating new DGC components. Establishment of the molecular composition of plasma-membrane protein complexes has been hampered by a lack of suitable biochemical approaches. Here we present an analytical workflow based upon the principles of protein correlation profiling that has enabled us to model the molecular composition of the DGC in mouse skeletal muscle. We also report our analysis of protein complexes in mice harboring mutations in DGC components. Bioinformatic analyses suggested that cell-adhesion pathways were under the transcriptional control of NFκB in DGC mutant mice, which is a finding that is supported by previous studies that showed NFκB-regulated pathways underlie the pathophysiology of DGC-related muscular dystrophies. Moreover, the bioinformatic analyses suggested that inflammatory and compensatory mechanisms were activated in skeletal muscle of DGC mutant mice. Additionally, this proteomic study provides a molecular framework to refine our understanding of the DGC, identification of protein biomarkers of neuromuscular disease, and pharmacological interrogation of the DGC in adult skeletal muscle https://www.mda.org/disease/congenital-muscular-dystrophy/research.

The muscular dystrophies are hereditary diseases characterized primarily by the progressive degeneration and weakness of skeletal muscle. Most are caused by deficiencies in proteins associated with the cell membrane (*i.e.* the sarcolemma in skeletal muscle), and typical features include instability of the sarcolemma and consequent death of the myofiber (1).

One class of muscular dystrophies is caused by mutations in genes that encode components of the sarcolemmal dystrophin-glycoprotein complex (DGC). In differentiated skeletal muscle, this structure links the extracellular matrix to the intracellular cytoskeleton. The DGC consists of dystroglycan (DG)^1^, the sarcoglycan-sarcospan complex, dystrophin (DMD), and dystrophin-associated proteins. In its mature form, the DG component is comprised of a cell-surface-associated alpha subunit (α-DG) and a transmembrane beta subunit (β-DG) (2). Whereas α-DG functions as receptor for extracellular proteins (3), β-DG binds dystrophin via its cytoplasmic domain and thereby links the actin cytoskeleton to the plasma membrane (4). The dystrophin-associated proteins are alpha-dystrobrevin (DTNA), alpha-syntrophin-1 (SNTA1), beta-syntrophin-1 (SNTB1), and neuronal nitric oxide synthase (NNOS). Further stabilization of the DG-axis occurs through thesarcoglycan-complex, which consists of five subunits: alpha-sarcoglycan (SGCA), beta-sarcoglycan (SGCB), delta-sarcoglycan (SGCD), gamma-sarcoglycan (SGCG), and Sarcospan (SSPN) (5).

A common feature of the DGC-related muscular dystrophies is loss or severe reduction of the entire DGC, because of genetic abnormalities of a single component (6–8). This general loss of proteins leads to a pathogenic mechanism that is hypothesized to account for the significant overlap in pathological features of numerous muscular dystrophies. Although gene-expression profiling has been used to identify the common pathogenic mechanisms underlying disease in the context of DGC mutations (9), this approach failed to provide a deeper understanding of the structural interface between the extracellular matrix and the cytoskeleton, because no inferences could be made about the cellular localization of the proteins affected by differential gene expression.

A complete molecular understanding of communication between the extracellular matrix and intracellular environment via integral membrane proteins on the cell surface requires comprehensive biochemical characterization of the plasma-membrane proteome. However, despite recent advances in proteomic approaches, the analysis of intact membrane-protein complexes continues to pose technical difficulties (10). First, the experimental conditions under which particular membrane protein complexes can be extracted and preserved must often be determined empirically. Second, integral membrane proteins are inherently insoluble and notoriously difficult to manipulate and characterize biochemically. Third, tandem mass spectrometry (MS)-derived polypeptide sequences typically exclude the mostly hydrophobic amino acids that comprise the transmembrane domains of integral membrane proteins (11); typically, highly concentrated acids (*e.g.* formic acid) or volatile aqueous/organic solvent mixtures (*e.g.* acetonitrile) are required for the analysis of membrane proteins by such approaches (12, 13).

Several proteomic strategies have been used to detect differentially expressed integral membrane proteins involved in muscular dystrophy. The predominant method has been 2-dimensional gel electrophoresis (2DGE) followed by in-gel trypsin digestion and tandem MS (MS/MS) (14–16). More recently, 1-dimensional gel electrophoresis (1DGE) has been utilized in the characterization of normal, aging, and dystrophic skeletal muscle (17–19). Although differentially expressed proteins in muscular dystrophy samples were identified using the 2DGE proteomic strategy, a number of technical shortcomings make the use of this approach impractical for the study of integral membrane proteins by MS/MS. First, the low resolving power and limited dynamic range of the 2DGE method make it difficult to separate complex protein samples and acquire MS/MS data on low-abundance integral membrane proteins that may be present in the sample; this shortcoming is borne out by the underrepresentation of this class of proteins in data sets from such analyses (20). Second, as stated above, integral membrane proteins are poorly resolved by 2DGE because they are insoluble (21). Although recent improvements in sample preparation have made the 2DGE method more amenable to the proteomic analysis of skeletal muscle (*e.g.* biochemical fractionation removing high-abundance contractile proteins from crude muscle extracts facilitated proteomic analysis of low-abundance membrane proteins by the 2DGE strategy (22)), this approach did not improve the detection of integral membrane proteins.

Nongel based proteomic approaches have gained in use over the past years and also been used to study the DGC in muscular dystrophy. For example, DGC components were directly immunoprecipitated and the samples were then biochemically analyzed using shotgun proteomic methods (23–25). Although this approach identified many components of the DGC, and even putative novel interacting proteins, the scale of this proteomic analysis was relatively small. Moreover, the antibody used to immunoprecipitate the DGC might have influenced the stability of the complex, and thus the proteomic results likely depended on both antigen and antibody. A second approach was based on analysis of soluble protein fractions from skeletal muscle by label-free, reverse-phase liquid chromatography followed by MS/MS (26). In a third (and similar) approach, a label-free shotgun proteomic analysis was used to analyze cardiac muscle from the dystrophin-deficient (*mdx*) and WT mice, revealing differential expression of a number of proteins involved in stabilizing the basal lamina and organizing the cytoskeleton (27).

Recent advances in mass spectrometer instrumentation (*i.e.* linear quadrupole-Orbitrap mass analyzer) in conjunction with optimized sample preparation workflows (*i.e.* isoelectric peptide focusing OffGel fractionator) and software platforms (*i.e.* MaxQuant) has facilitated the in-depth proteomic analysis of a skeletal muscle cell line, skeletal muscle tissue, and single muscle fibers using label-free shotgun proteomic acquisition schemes (28, 29). Similar label-free shotgun proteomic workflows have recently identified global proteomic changes in contraction, energy metabolism, extracellular matrix, and cytoskeleton of skeletal muscle of *mdx* mice (16). Moreover, interfacing differential subcellular protein fractionation of skeletal muscle tissue with the label-free shotgun approach uncovered a reduction in full-length dystrophin isoform Dp427 expression and perturbations in the expression of protein networks involved in metabolism, signaling, contraction, ion-regulation, protein folding, the extracellular matrix, and the cytoskeleton in *mdx^4cv^* mice (15). Despite these findings, the proteomic identification of the DGC expressed in skeletal muscle remains incomplete to date.

In this report, we present a novel biochemical workflow for the isolation, identification, and mass spectrometry-based quantification of protein complexes on the plasma membrane of mouse skeletal muscle, and report on several important discoveries related to muscular dystrophy using this workflow. Using lectin (wheat-germ)-affinity chromatography in combination with sucrose-gradient fractionation to isolate such protein complexes, we carried out MS/MS-based proteomic analysis of the DGC in both WT mice and mouse models of DGC-related muscular dystrophy. Specifically, label-free, directed mass spectrometry analysis (dMS) (30, 31) of proteins fractionated by sucrose-gradient centrifugation validated the comigration of components of the DGC in skeletal muscle from WT mice, but not the dystrophin-deficient *mdx* and delta-sarcoglycan-null (Sgcd-null) models of muscular dystrophy (32, 33). Moreover, comparative analysis of protein expression between the WT and DGC-mutant mice revealed transcriptional up-regulation of a protein network involved in cell adhesion in the DGC-mutant mice, strongly suggesting that compensatory cell adhesion network(s) are activated in these models of muscular dystrophy. In summary, our biochemical workflow effectively interrogated the composition of the DGC and DGC-related muscular dystrophies in skeletal muscle, and also provides an experimental strategy to validate novel DGC components in skeletal muscle using targeted proteomic methods.

## EXPERIMENTAL PROCEDURES

### 

#### 

##### Animals

Animal care, ethical usage, and procedures were approved and performed in accordance with the standards set forth by the National Institutes of Health and the Animal Care Use and Review Committee at the University of Iowa. C57BL6/J and *mdx* mice were obtained from The Jackson Laboratory. The Sgcd-null mice were developed in the Campbell laboratory as described elsewhere (33).

##### Immunohistochemistry

Histopathological studies were performed as before (33, 34). Polyclonal antibodies against DMD (Abcam, Cambridge, UK) and SGCD (R214), and monoclonal antibodies against α-DG (IIH6), β-DG (AP83), SGCA (20A6), and SGCG (21B5) were used for immunohistochemistry.

##### Sucrose Density Gradient Biochemistry

Skeletal muscle was isolated from adult mice, and microsomes were prepared as described elsewhere (35). Nonwashed microsomes were solubilized overnight at 4 °C while rotating in a solution of 1% digitonin, 50 mm Tris-HCl pH 7.4, 150 mm NaCl, and proteinase inhibitors. Nonsolubilized proteins were pelleted at 100,000 × *g*. The supernatant was incubated overnight with wheat-germ agglutinin (WGA)-agarose beads (Vector Laboratories, Burlingame, CA) at 4 °C. The beads were washed 3x in washing buffer (0.1% digitonin, 50 mm Tris-HCl pH 7.4, 150 mm NaCl, proteinase inhibitors), and proteins were eluted by 1-hour incubation with 0.3 m N-acetyl-d-glucosamine in washing buffer, at 4 °C. The eluted proteins were then fractionated by centrifugation (2 h at 238,000 × *g*) through a 5–30% sucrose gradient. Fractions were collected from the top of the gradient and analyzed by SDS-PAGE.

##### Coimmunoprecipitation Biochemistry and Immunoblotting

Skeletal muscle was isolated from rabbit skeletal muscle (Pelfreeze, 41225–2), and microsomes were prepared as described elsewhere (4). Total microsomes were solubilized overnight at 4 °C while rotating in a solution of 1% digitonin, 50 mm Tris-HCl pH 7.4, 150 mm NaCl, and proteinase inhibitors. Nonsolubilized proteins were pelleted at 100,000 × *g*. The supernatant was incubated overnight with wheat-germ agglutinin (WGA)-agarose beads (Vector Laboratories) at 4 °C. The beads were washed 3x in washing buffer (0.1% digitonin, 50 mm Tris-HCl pH 7.4, 150 mm NaCl, proteinase inhibitors), and proteins were eluted by 1-h incubation with 0.3 m N-acetyl-d-glucosamine in washing buffer, at 4 °C. Buffer exchange (0.1% digitonin, 50 mm Tris-HCl pH 7.4, 150 mm NaCl, and proteinase inhibitors) was performed to remove N-acetyl-d-glucosamine. Antibody beads were generated by coupling ∼3.5 mg of anti-dystroglycan antibody (IIH6), anti-ryanodine receptor antibody (XA7), or bovine serum albumin (BSA) to Sepharose beads using activated CNBr according to manufacturer's protocol (GE Healthcare Life Sciences, Cambridge, United Kingdom). WGA-enriched proteins were incubated with antibody beads, followed by washing 3× (0.1% digitonin, 50 mm Tris-HCl pH 7.4, 150 mm NaCl, proteinase inhibitors), and eluted in 0.1 m triethylamine, pH 11.5. Western blots were incubated with antibodies against α-DG and β-DG (AF6868), DMD (Abcam), SGCA (20A6), SGCB (G26), SGCG (21B5), SGCD (R214), ADBN (Transduction Laboratories, San Jose, CA), and NNOS (R200) as described elsewhere (36).

##### Experimental Design and Statistical Rationale

##### Processing of MS Samples

Using large-scale filter assisted sample preparation, samples were buffer exchanged four times with 8 m Urea, 50 mm Tris-HCl, pH 8.5, and 100 mm beta-mercapto-ethanol. The first three rounds of buffer exchange were performed for 2 h, and the fourth overnight. Next, samples were concentrated (Amicon Ultra, 10 kDa cut-off MW, Millipore, Billerica, MA) and separated by SDS-PAGE on 4–12% gels (Invitrogen) for analysis by silver staining. Samples were reduced with 10 mm dithiothreitol (DTT) at 37 °C in 8 m Urea, 50 mm Tris-HCl, pH 8.5 for 30 min, and subsequently alkylated with 55 mm iodoacetamide for 30 min in the dark. Samples were diluted to a final concentration of 0.5 m urea with 50 mm Tris-HCl pH 8.5. Each sample was treated with 1 μg of sequence-grade trypsin (Promega, Sunnyvale, CA) and incubated overnight at 37 °C. Samples were then spiked with a tryptic digest of bovine serum albumin (BSA, Fremont, CA) containing iodoacetic-acid alkylated cysteine residues (Michrom Bioresources, Auburn, CA), at a 1:75 molar ratio. Samples were acidified and desalted on Vydac C18 spin-columns (The Nest Group), and then subjected to SCX fractionation on polysulfoethyl-A packed spin-columns (The Nest Group) according to the manufacturer's protocol. Briefly, desalted samples were dissolved in SCX buffer B (5 mm KHPO_4_, 25% acetonitrile (ACN, Southborough, MA)) and loaded onto SCX spin-columns, and the tryptic digests were then released from the SCX spin-columns using a three-step KCl elution gradient developed from a mixture of buffer B and buffer C (5 mm KHPO_4_, 25% ACN, 350 mm KCl). Salt-eluted fractions were desalted, dried down, and dissolved in MS loading buffer (1% acetic acid, 1% ACN). A biological replicate *n* = 1 for the proteomic comparison between wt, mdx, and Sgcd mouse models. The application of orthogonal protein fractionation methods (*i.e.* sucrose gradient density centrifugation, and strong-cation exchange peptide chromatography) coupled with the directed mass spectrometry workflow facilitated an in-depth proteomic comparison of skeletal muscle tissue between wildtype and muscular dystrophy mouse models. WGA-enriched trypsin-digested samples from each mouse strain were subjected to spin-column strong cation exchange chromatography. Each salt-bumped SCX fraction was subjected to in-depth proteomic coverage using the directed MS workflow. The iodoacetic-acid, trypsin-digested BSA protein standard was spiked into each protein sample to provide independent markers to detect systematic errors within and between independent samples. The spiked standard also provided a method to detect systematic errors in mass spectrometry instrumentation that could skew the proteomic findings. The proteomic findings for each mouse strain were derived from pooled skeletal muscle samples representing 10–15 individual mice from the wildtype, mdx, or Sgcd mouse colonies.

##### Mass Spectrometry

The samples were subjected to LC-MS/MS on the 6520 Agilent Accurate-Mass Quadrupole Time-of-Flight (Q-TOF) mass spectrometer, interfaced with the HPLC Chip Cube. The samples were loaded onto the large-capacity C18 Chip II (160 nL enrichment column, 9 mm analytical column) and subjected to LC-MS/MS analysis for 90 min in a gradient of 1.5% to 35% buffer B (100% ACN, 0.8% AA). Full MS (MS1) data were acquired with a mass range of 400–1250 m/z and an acquisition rate of 1 spectra/second. From these data, an ion preference list was generated using Agilent MassHunter Qualitative Software. dMS was performed using the following settings: a maximum of 10 ions per cycle, a narrow isolation width (∼1.3 atomic mass units), precursor masses dynamically excluded for 30 s after 8 MS/MS in a 30-s time window, and use of the preferred ion list. The capillary voltage and capillary temperature settings for MS were set to 1800 V and 330 °C, respectively. The infused reference mass of 1221.9906 was used to correct precursor m/z masses in each LC-MS/MS experiment.

##### Protein Identification

The raw.d files were searched against the UniProt mouse database (downloaded April 18th, 2012; with 55190 entries) using SpectrumMill Software version B.04.00.127 and the following settings: precursor mass tolerance of 25 parts per million (ppm), product mass tolerance of 200 ppm, and a maximum of two trypsin miss cleavages. Searches for post-translational modifications included a static carbamidomethylation on cysteine residues (C = 57.02146 AMU), differential modifications for oxidized methionine (M = 15.9949 AMU), phosphorylated serine, threonine, tyrosine (STY = 79.9663 AMU), and ubiquitinated lysine (K = 114.0429 AMU). For normalization between the samples, the raw.d files were searched against the UniProt bovine database with same search settings as above except the files were searched with a static carboxymethylation on cysteine residues (C = 58.005 AMU). A total of 14 cysteine-containing BSA peptides were quantified and the total intensities of these peptides were collated for each sample and the differences between samples were normalized (supplemental Table S1). The peptide hits were autovalidated with the “Fixed thresholds” setting using the following values: Score Threshold of 3, % Spectral Intensity (SPI) Threshold of 30, and Rank 1-Rank 2 Threshold of 2, with delta Fwd-Rev not checked such that the initial False-Discovery Rate (FDR) was ≤ 7%. To reduce the FDR well below ∼7% the proteomic results were further filtered by applying a delta Fwd-Rev score of 1.2 to provide the most stringent filter in the removal of false positive protein identifications. When there were distinct peptides that uniquely represent multiple protein isoforms, the individual member is reported, and the quantification was based on the mean of the peptide spectrum matches (PSM) for that specific isoform.

##### Data Deposition

The mass spectrometry proteomics data have been deposited to the ProteomeXchange Consortium (http://proteomecentral.proteomexchange.org) via the PRIDE partner repository with the data set identifier <PXD004020>. The annotated spectra are accessible through the MS-Viewer module at http://prospector2.ucsf.edu/prospector/cgi-bin/msform.cgi?form=msviewer. Key: 61ze1ebl1o.

##### Network Visualization and Public Protein Interaction Databases Used

Protein interaction networks were visualized using Cytoscape 3.1.0 (http://www.cytoscape.org) (37). Interactions were identified using the GeneGo bioinformatic software (http://www.genego.com).

##### Functional Analyses

Gene ontology analyses were performed using the “WebGestalt” software package (38). Cluster analysis was performed using the “Cluster” software package (39). K-means clusters were calculated based on absolute correlation (centered).

##### Quantitative RT-PCR

Total RNA was isolated from skeletal muscle from WT, Sgcd-null, and *mdx* mice using TRIzol (Ambion, South San Francisco, CA) according to the manufacturer's protocol. Five micrograms of total RNA was treated with DNase I (Promega), and then reverse transcribed using AMV reverse transcriptase (Roche, Basel, Switzerland) according to the manufacturer's protocol. qRT-PCR was performed using RT2 qPCR primers (Qiagen, Hilden, Germany) against Itga5, Itga6, Cd151, and Vav1. Reactions were performed on a Bio-Rad iCycler. Statistical analyses were performed as described elsewhere (40).

## RESULTS

### 

#### 

##### Isolation and Separation of N-linked Glycosylated Proteins from Skeletal-muscle Microsomes

**Fig. 1.**
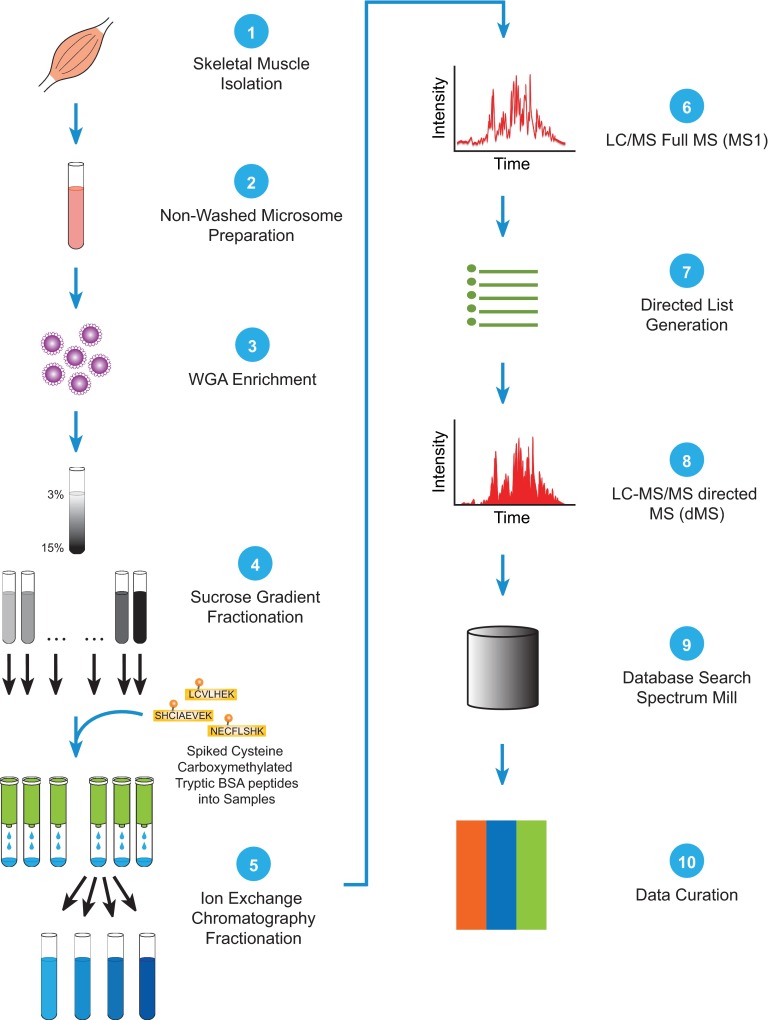
**Schematic illustration of workflow.** Skeletal muscle is dissected (1) and subjected to subcellular fractionation to enrich for membrane proteins (2). Glycoproteins and associated proteins are isolated using wheat-germ agglutinin (WGA) chromatography (3), and are separated by sucrose-gradient fractionation (4). Fractions of interest are spiked with cysteine carboxymethylated tryptic BSA peptides and subjected to ion-exchange chromatography to reduce sample volume; they are eluted using increasing salt concentrations (5). A first LC/MS run is performed on each elution fraction (6), and the peptides identified are used to generate a directed list (7). A directed LC-MS/MS run is then performed to provide quantitative data (8). Proteins are annotated based on queries of the Spectrum Mill database (9). The annotated quantitative data is then curated (10).

We developed a comparative proteomic workflow and applied it to skeletal muscle tissue from WT, *mdx*, and Sgcd-null mice. This consisted of the following steps (also summarized in Fig. 1). First, nonwashed microsomes were prepared from muscle isolated from WT, *mdx*, and Sgcd-null mice, and solubilized in 1% digitonin. These samples were subjected to lectin-affinity chromatography using wheat-germ agglutinin, and then eluted with N-acetyl glucosamine. Next, the samples were layered on a 5–30% sucrose gradient and subjected to centrifugation. Select sucrose-density gradient fractions (4–8) were subjected to large-scale, filter-aided sample preparation (FASP) and digested with trypsin (41). The C18-desalted peptide samples were further fractionated by strong-cation exchange (SCX) spin-column chromatography, and then subjected to high-resolution LC-MS to generate a preferred list of 2+ and 3+ peptide ions. Select samples were then subjected to directed mass spectrometry, and the resulting data files were searched with the Spectrum Mill program for nonredundant protein identifications (IDs).

Relative quantification of proteins across sucrose fractions was made possible by spiking each FASP sample with approximately ∼250 picomoles of alkylated (using iodoacetic-acid) trypsin-digested bovine serum albumin (BSA). This step was performed prior to the fractionation of tryptic peptides by SCX spin-column chromatography, so that BSA peptides (in particular alkylated-cysteine BSA peptides) would cofractionate with sample tryptic peptides based on their charge state. The +1 atomic mass unit (AMU) differential between iodoacetic acid (*i.e.* MW-+58, carboxymethyl) and iodoacetamide (*i.e.* MW-+57) in the spiked samples makes it possible to distinguish between peptides containing cysteines with iodoacetic-acid or iodoacetamide. Trypsin-digested iodoacetic-acid labeled BSA was selected as the external standard because it incorporates 26 tryptic cysteine-containing peptide ions (+2, +3 peptide ions, *m*/*z* window of 400–1250) that are fractionated based on their charge state during SCX spin-column chromatography. Subjecting the desalted peptide samples to high-resolution LC-MS on the Agilent 6520 Quadrupole Time-of-Flight high-resolution mass spectrometer yielded a list of preferred peptide ions, and these were targeted for MS/MS sequencing using a dMS protocol and the MassHunter data acquisition software. Pilot proteomic experiments demonstrated that the dMS acquisition protocol was superior to the traditional data-dependent acquisition (DDA) protocol in the identification and quantification of trypsin-digested bovine serum albumin on the 6520 QTOF (Supplemental Table 2). This label-free analytical workflow was expected to facilitate the relative quantification of proteins in muscle samples, and to provide a platform for monitoring and detecting systematic errors that would skew the relative protein quantification between samples. Sources of systematic error might include reagent failure (*i.e.* corrupted SCX and/or C18 spin columns), differences in sample handling (*e.g.* pipetting error), and changes (*e.g.* decreases) in sensitivity of the mass spectrometer.

**Fig. 2.**
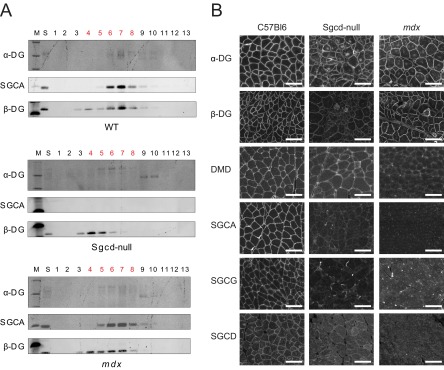
**Disruption of the dystrophin-glycoprotein complex in DGC mutants.** *A*, Sucrose-gradient sedimentation was used to analyze protein complexes in wheat-germ agglutinin-enriched, nonwashed microsomes from WT, Sgcd-null and *mdx* mice. A 5–30% sucrose gradient was run. Light-to-heavy fractions (1–13) were separated by SDS-PAGE, and the expression of α-DG, SGCA, and β-DG was detected by immunoblotting. Fractions depicted in red were subjected to proteomic analysis. *B*, Immunohistochemistry was used to detect DGC subunits in skeletal muscle cryosections from WT, Sgcd-null, and *mdx* mice.

We next determined whether skeletal muscle from WT, *mdx*, and Sgcd-null mice subjected to lectin-affinity chromatography and sucrose-gradient fractionation expressed the DGC at detectable levels, as this was critical to quantitative proteomic analysis (Fig. 1). Western blot analysis of DGC subunits α-DG, SGCA, and β-DG in the WT samples revealed that all three proteins were detectable in sucrose fractions 6–8 (Fig. 2*A*, top panel). In the *mdx* sample, the migration pattern was broader, with each subunit detectable across sucrose fractions 4–10 (Fig. 2*A*, bottom panel). In the Sgcd-null sample, where SGCA was not detectable in any fraction, α-DG was detectable across fractions 5–10, and β-DG was restricted to fractions 3–6 (Fig. 2*A*, middle panel). Measurement of sucrose densities per fraction confirmed equal gradients for each mouse model (supplemental Fig. S1). The demonstration of co-fractionation of the DGC subunits in the WT sample supports the notion that the DGC is normally a stable higher MW complex in skeletal muscle. The discordant migration of DGC subunits through the sucrose gradient in *mdx* and Sgcd-null mice suggests that the complex was perturbed in these contexts (Fig. 2*A*). Indeed, visualization of DGC subunits using immunohistochemistry showed that the DGC is localized at the plasma membrane in WT mice (Fig. 2*B*). Conversely, in mdx and Sgcd-null mice the membrane expression of subunits of the DGC is lost, indicating loss of the DGC as a stable higher MW complex (Fig. 2*B*).

##### Quantitative Proteomic Analysis of Microsomes from WT, Sgcd-null, and mdx Skeletal Muscle

**Fig. 3.**
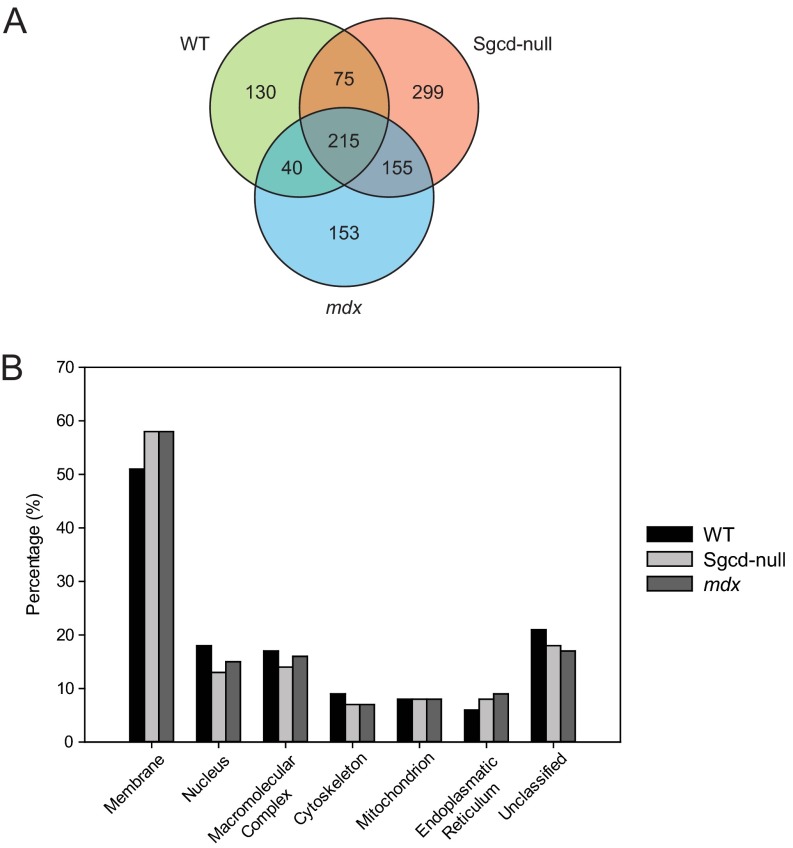
**Classification of data set.** *A*, A Venn diagram shows the distribution and number of proteins detected in microsomes from WT mice and the Sgcd-null and *mdx* disease models. *B*, Bar graphs showing the percentages of the top six classifications for the combined protein expression data per mouse model listed below, along with the group “Unclassified.” For each gene ontology term, the percentage refers to the fraction of classified proteins per total number of proteins (all classes).

Sucrose-gradient fractions 4–8 from WT, *mdx*, and Sgcd-null skeletal muscle were subjected to quantitative mass spectrometry (summarized in Fig. 1). dMS analysis of these samples resulted in 460, 563, and 744, respectively, nonredundant protein IDs. As shown in the Venn diagram in Fig. 3*A*, 215 proteins were shared among all three samples, 40 between the WT and *mdx* samples, 75 between the WT and Sgcd-null samples, and 155 between the *mdx* and Sgcd-null samples. A further 130, 153, and 299 proteins were restricted to the WT, *mdx*, and Sgcd-null skeletal muscle samples, respectively. In total, 1067 proteins were identified across the WT, Sgcd-null and *mdx* samples (supplemental Table S5).

We next evaluated the cellular distributions of the identified proteins to determine if lectin-affinity chromatography had sufficient power to enrich for proteins in, and associated with, the skeletal-muscle membrane. To this end, we uploaded the proteins identified in the WT, Sgcd-null, and *mdx* samples into the WEB-based GEne SeT AnaLysis Toolkit (WebGestalt) and searched the Gene Ontology (GO) annotations (38). This analysis indicated that, for each genotype, more than 50% of the identified proteins are membrane related (WT 51%, Sgcd-null 58%, and *mdx* 58%). The remaining proteins identified in each sample were either unclassified or annotated as components of the nucleus, macromolecular complexes, cytoskeleton, mitochondrion, or endoplasmic reticulum. Interestingly, although the number of proteins identified in the samples from Sgcd-null and *mdx* mice was higher than those in WT mice, the overall distributions (percentages in various membranes within the cell) were similar (Fig. 3*B*). These findings confirm that lectin-affinity chromatography was effective in enriching for membrane and membrane-associated glycoproteins.

**Fig. 4.**
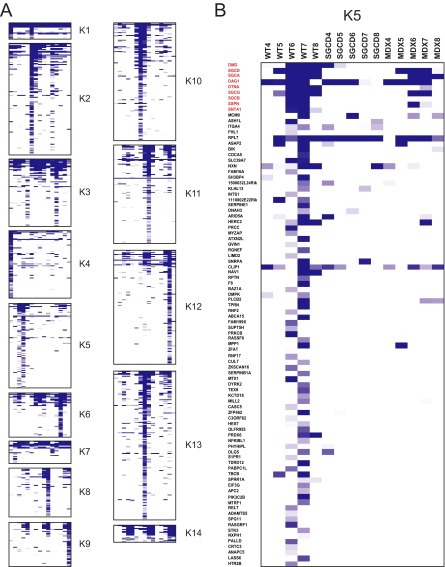
**K-means clustering.** *A*, Partitioning of the 1067 proteins detected in our screen into 14 clusters using K-means clustering. In all cases, the columns represent sucrose-gradient fractions from muscle taken from WT, Sgcd-null, and *mdx* mice (*n* = 5 per genotype). Heatmap intensities represent relative protein expression. *B*, Expanded view of K-means cluster 5 (K5), which contains known DGC components (red labels).

Our results prompted us to implement an unbiased strategy for detecting groups of proteins that co-migrate across the sucrose-density gradient, with the aim of identifying complexes that form in the membranes of skeletal muscle. To this end, we applied empirical *K*-means clustering to the proteomic data sets, partitioning them based on their pattern of migration through the sucrose density gradient. This approach identifies putative protein complexes based on the principle that proteins belonging to a complex will behave similarly, and it resulted in the grouping of 1067 nonredundant IDs from the three genotypes into 14 protein clusters (Fig. 4*A*).

To determine whether each of the 14 protein clusters is linked to specific biological processes that occur in the skeletal muscle, we uploaded them individually into the *GeneGo* bioinformatics software program. The top-ranked biological process networks associated with each cluster were identified (supplemental Table S3). As expected based on the regeneration processes that are active in muscular dystrophy, the top-ranked process network was development/neuromuscular junction, and was associated with cluster K5 (*p* = 2.88E^−11^). Other highly ranked biological process networks included cell-adhesion/platelet-endothelium-leukocyte interactions for cluster K10 (*p* = 1.46E^−10^), and cell adhesion/cell matrix-interactions for cluster K11 (*p* = 6.24E^−10^). Notably, the proteins in clusters K10 and K11 were identified mainly in the Sgcd-null and *mdx* mice, consistent with their up-regulation in muscular dystrophy. Overall, this bioinformatic analysis identified biological process networks (in specific protein clusters) that are related to the physiology of skeletal muscle tissue in WT, Sgcd-null, and *mdx* mice.

Our results allowed for the development of a molecular framework to examine known components of the DGC, and possibly identify novel DGC components in skeletal muscle. To this end, we generated physical protein-protein interaction (PPI) maps using the *GeneGo* software, applying the “shortest path” algorithm and taking only direct binding interactions into account for each of the 14 clusters (supplemental Fig. S3). Close inspection of these PPI maps revealed that the DGC was part of cluster K5 (supplemental Fig. S5). This cluster is characterized by the co-migration of proteins in sucrose-gradient fraction 7 of the WT sample (Fig. 4*B*). The DGC components in this cluster contained dystroglycan (listed as DAG1), dystrophin (DMD), SGCA, SGCB, SGCD, SGCG, SSPN, SNTA1, and DTNA. Notably, several of these DGC components were visibly reduced in the *mdx* sample, and most were undetectable in the Sgcd-null sample. These results were concordant with the Western blot and immunohistochemistry results in skeletal muscle between wt, *mdx*, and Sgcd-null mice (Fig. 2).

**Fig. 5.**
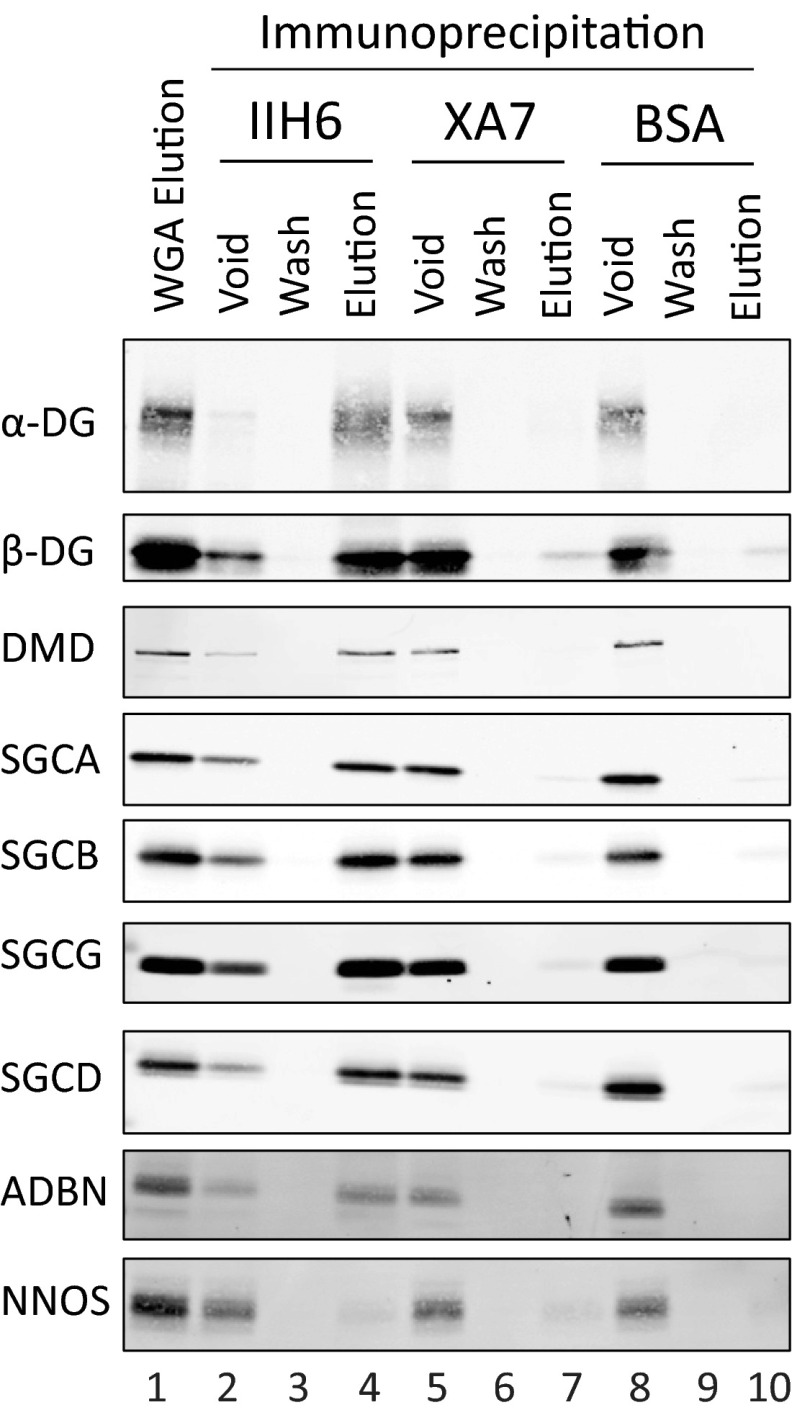
**Co-immunoprecipitation of DGC subunits.** Lane 1, input; lanes 2–4, anti-alpha-dystroglycan beads (IIH6); lanes 5–7, anti-ryanodine receptor beads (XA7); lanes 8–10, bovine serum albumin beads (BSA). Western blot analysis of α-DG, β-DG, DMD, SGCA, SGCB, SGCG, SGCD, ADBN, NNOS.

The comigration of proteins through a sucrose density gradient does not provide definitive proof that the proteins interact as a protein complex, thus we performed a co-immunoprecipitation experiment to test if the detergent-solubilized DGC formed a macromolecular protein complex in solution. α-DG (IIH6) antibody-coupled Sepharose beads were generated alongside negative control beads composed of Ryanodine Receptor antibody (XA7) (*i.e.* negative control IgM antibody) or Bovine Serum Albumin (BSA) for the immunoprecipitation experiments. WGA-enriched rabbit skeletal muscle microsomes were subjected to immunoprecipitation to test if DGC subunits were specifically bound to IIH6 (Fig. 5). As predicted, the DGC subunits specifically bound to IIH6, whereas no DGC subunits were detectable in the negative control immunoprecipitations (Fig. 5, compare lane 4 to lanes 7 and 10).

These results allowed for the development of a molecular framework to examine known components of the DGC, and to identify novel DGC components in skeletal muscle. To this end, we generated a more comprehensive PPI map of the DGC-containing K5 cluster using the *GeneGo* bioinformatics software, again applying the shortest path algorithm but including only those direct interactions that involve maximally two nodes (supplemental Fig. S4). The proteins in cluster K5 that were identified in both this and the original analysis are depicted in blue, whereas the proteins depicted in gray represent “missing links.” Known DGC components are shown with red borders, and proteins that bind directly to them with green borders. Inspection of this PPI map revealed proteins that are mutated in muscular dystrophies and had not previously been linked to the classical DGC (42, 43). For example, plectin1 (PLEC1) is linked to several DGC components, and also to MYZAP, mutant forms of which are associated with severe skeletal-muscle dysfunction (44). Mutant forms of desmin (DES) also cause muscular dystrophy, and our analysis associated this protein with the DGC for the first time. Other known DGC-interacting proteins identified include utrophin (UTRN), growth factor receptor-bound 2 (GRB2), and neuronal nitric oxide synthase (NNOS) (45). The PPI map also included phospholipase C beta2 (PLCB2), which can bind to DTNA or SNTA1 (46, 47). Notably, PLCB2 was recently shown to regulate calcium influx into skeletal muscle, and this regulation was abrogated in muscular dystrophy (47). Alpha-neurexin, which is present at the neuromuscular junction (NMJ) and is a known DG ligand (48), also formed an edge to DG. Overall, this PPI map demonstrates that the K5 protein cluster includes known components of the DGC, and can serve as a platform for examining putative DGC-interacting proteins as candidates for novel muscular dystrophy-causing genes.

**Fig. 6.**
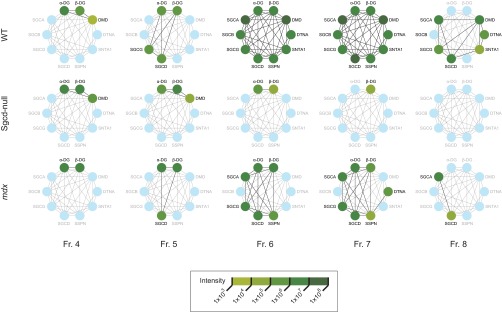
**Quantitative analysis of expression of DGC components.** Intensities of expression of DGC components present in cluster K5, for sucrose-gradient fractions 4–8 from the WT, Sgcd-null and *mdx* mice.

We manually constructed a force-directed graph of the DGC to facilitate more in-depth quantification of the DGC proteins in the K5 protein cluster (Fig. 6). For the purposes of this analysis, DG expression is represented as separate expression of the α-DG and β-DG subunits. When the WT, Sgcd-null, and *mdx* samples were compared, the intensity of DGC components ranged across four orders of magnitude. For example, whereas in the WT samples all DGC components were present in sucrose fractions 6 and 7 (with the intensity for all components being between 1 × 10^6^ and 1 × 10^8^), in the *mdx* samples the DMD, SNTA, and SGCG subunits were completely undetectable, and the levels of others were reduced (*e.g.* SSPN in fraction 7 at 1 × 10^3^ *versus* 1 × 10^6^). Moreover, in the Sgcd-null sample, only the α-DG and β-DG subunits of the DGC were detectable, and at levels significantly lower than in the WT sample (*e.g.* β-DG in fraction 7 at 1 × 10^3^ *versus* 1 × 10^6^), corroborating earlier findings indicating that the skeletal-muscle DGC requires SGCD expression. Overall, these quantitative proteomic results provide strong evidence that the DGC is compromised in mouse models of muscular dystrophy, validating the results from earlier biochemical and immunohistochemical analyses.

##### Putative Compensatory Cell-adhesion Networks Identified in Mouse Models of Muscular Dystrophy

Our lack of insight into the regulatory processes that lead to shared pathological features of DGC-related muscular dystrophies (49) is a significant gap, and our comparative proteomic analysis of membrane protein complexes in WT, Sgcd-null, and *mdx* skeletal muscle provided a unique opportunity to address this. For example, *K*-means clustering revealed that the expression of protein clusters K10 and K11 was increased in both the mutant *versus* the WT samples. This preliminary finding prompted us to examine in greater depth which protein(s) and protein networks might represent biomarkers of disease processes that underlie the skeletal muscle pathology in dystrophies related to DGC dysfunction. First we performed *GeneGo* transcriptional network analysis on proteins whose expression was elevated at least twofold in the Sgcd-null or *mdx versus* WT samples. This analysis had the potential to identify transcription factors that globally regulate protein expression in skeletal muscle.

**Fig. 7.**
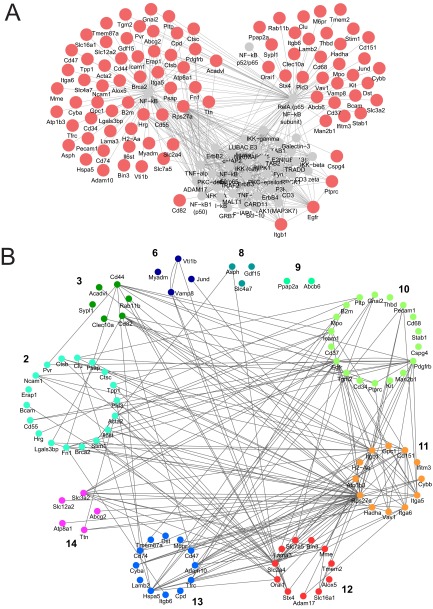
**NFκB-regulated protein expression.** *A*, NFκB-responsive proteins that are up-regulated twofold and more in mouse models of muscular dystrophy are shaded in red. Proteins that are associated with the network according to *GeneGo* are shaded in gray. *B*, NFκB-regulated proteins sorted according to *K*-means cluster.

A total of 302 proteins were subjected to this analysis, and the top-ranked subnetwork centered on NFκB (Supplemental Table 4). The NFκB (RelA-p65)-centric network consisted of 129 nodes and 478 edges, including 91 of the 129 nodes identified in the proteomic screen (Fig. 7*A*). The identification of this network was notable in light of two other observations: patients afflicted with Duchenne muscular dystrophy commonly exhibit NFκB-mediated inflammatory morbidity, for which they are treated with glucocorticoids; and the recently developed anti-inflammatory steroid VBP15 reduces muscular dystrophy in *mdx* mice, and does so by inhibiting the NFκB pathway, and does not cause the deleterious side effects of glucocorticoid-based therapeutics that are related to immune function (50).

We next set out to determine if the 91 proteins of the NFκB hub fall into specific clusters, which would establish whether NFκB-regulated proteins comigrate, *i.e.* are likely to form protein complexes, in both the Sgcd-null and *mdx* samples. Coordinated regulation of these protein complexes by NFκB might reflect disease processes that are active in both mouse models of muscular dystrophy. A PPI map of the 91 proteins identified 184 edges (Fig. 7*B*). Forty-eight of the proteins were restricted to clusters K2 (18 proteins), K10 (18 proteins), and K11 (11 proteins), networks whose functions are associated with cell-adhesion/cell-matrix and platelet/endothelium interactions (Fig. 7*B*, supplemental Table S3). Closer inspection of these protein clusters showed that integrins, which mediate adhesion between cells and the extracellular matrix, were up-regulated in both the Sgcd-null and *mdx* samples (Fig. 7*B*).

**Fig. 8.**
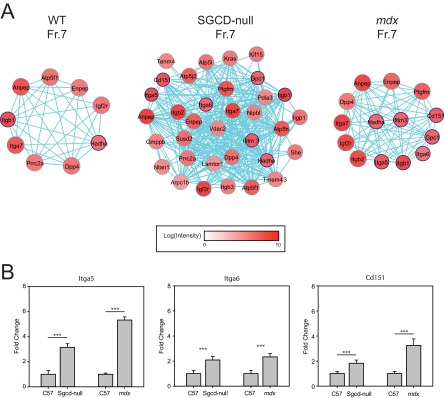
**Quantitative analysis of expression of components of the integrin-related network.** *A*, The integrin-related PPI map was generated based on cluster K10, using *GeneGo*. Intensities of expression are depicted for each protein detected in sucrose-gradient fraction 7 for the WT, Sgcd-null and *mdx* mouse models. *B*, Transcriptional up-regulation of NFκB-regulated genes in Sgcd-null and *mdx* mouse models detected by quantitative RT-PCR.

The finding of integrin up-regulation in the DGC mutant mice prompted us to examine the expression of these proteins in greater depth. Instability of the sarcolemma is a major pathology of skeletal muscle in DGC-mediated muscular dystrophies, and integrins have the potential to restore cell adhesion in skeletal muscle with this type of damage. We thus generated a force-directed graph of the proteins present in cluster K11, which contains the majority of the integrins that were identified in fraction 7 of the sucrose-density gradient in the *md*x and Sgcd-null samples, and identified the proteins regulated by NFκB (blue border) (Fig. 8*A*). These PPI maps consist of 9, 34, and 14 proteins in the WT, Sgcd-null, and *mdx* samples, respectively. Whereas the WT sample expressed only two integrins (itga7 and itgb1), the *mdx* sample expressed five (itga5, itga6, itga7, itgb1, and itgb2), and the Sgcd-null sample expressed six (all those expressed in *mdx* mice and ITGB3). Notably, the expression of ITGA5 and ITGA6 has been linked to active regeneration of skeletal muscle in muscular dystrophy (51), and both of these integrins were expressed only in the mutant samples (with the intensity for both components being between 1 × 10^6^ and 1 × 10^8^). Moreover, transgenic overexpression of ITGA7 reduces the skeletal-muscle pathology associated with dystrophin deficiency (52), and this integrin was highly up-regulated in both mutant samples (both at 1 × 10^7^ *versus* 1 × 10^6^ in WT). This finding supports the notion (52, 53) that ITGA7 can ameliorate cell adhesion defects in skeletal muscle in the context of muscular dystrophy. Expression levels of three NFκB-regulated genes (Itga5, Itga6, and Cd151) validated the increase in protein abundance (Fig. 8*B*). A total of 13 distinct integrins were detected across the three strains of mice (supplemental Table S6), where the majority of integrins (12 out of 13) showed either an increase in abundance or were only expressed in the mdx and Sgcd-null models (*i.e.* ITGAV, ITGA5, ITGA6, ITGAM, ITGB1, ITGB2, ITGB3, ITGA7, ITGAL, ITGB4, ITGAX, AND ITGB6). Overall, these data support a compensatory cell-adhesion model, whereby NFκB coordinates the up-regulation of integrin networks in response to perturbed DGC function in the context of muscular dystrophy.

##### Proteomic Profiling of Neuromuscular Disorders

**Fig. 9.**
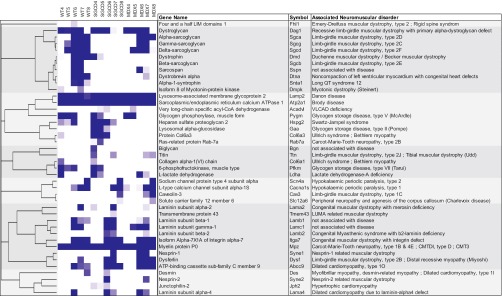
**Relationships among proteins associated with neuromuscular disorders.** Hierarchical clustering of the relative levels of protein expression per sucrose gradient fraction for WT, Sgcd-null and *mdx* mice. Proteins are grouped based on their similar migration patterns across mouse models, and similar distributions through the sucrose gradient.

The experimental approach presented here initially focused on proteomic analysis of biochemical fractions from distinct DGC-related mouse models and WT mice. Broadening the scope of our study by examining the levels of expression of proteins associated with other neuromuscular disorders, we found that 42 proteins of the proteins in our proteomic data set are listed in the “Gene Table of Neuromuscular Disorders”, a curated database for genes associated with neuromuscular diseases (54). Notably, hierarchical clustering of the 42 proteins across the WT, *mdx*, and Sgcd-null samples revealed that certain neuromuscular disorders group together (Fig. 9). Not surprisingly, given that the *mdx* and Sgcd-null models of muscular dystrophy were used for the comparative analysis, the DGC-related disorders clustered together. However, other types of neuromuscular disorders were also grouped; for example, glycogen-storage and related diseases (McArdle, Pompe, and Danon disease), as well as channel/transporter-related diseases (hypokalaemic periodic paralysis and Charlevoix disease). These findings were interesting on several levels. First, they suggest that certain biochemical pathways linked to muscular dystrophies that are unrelated to DGC defects are similarly affected in the *mdx* and Sgcd-null mouse models. Second, they indicate that pathways leading to different types of neuromuscular disorders are affected in, and shared by, various neuromuscular disorders. Therefore, interrogation of these proteins in the context of different neuromuscular disorders might lead to an understanding of the molecular relationships, as well as of some of the clinical similarities and phenotypes associated with certain neuromuscular disorders, although further research into the neuromuscular disease-associated pathways will be necessary to validate these hypotheses. Our findings are also interesting in that they suggest that the analysis of other mouse models of neuromuscular disease in a proteomic comparison like that presented here will expand our knowledge of, and clarify the relationships among, proteins that are involved in other neuromuscular diseases.

## DISCUSSION

### 

#### 

##### Proteomic Workflow

In this study we demonstrate a label-free proteomic workflow for studying the molecular composition of membrane protein complexes in mouse skeletal muscle, based upon the principles of protein correlation profiling and related proteomic studies (15, 16, 34, 40, 55). This workflow facilitated molecular characterization of the DGC in WT skeletal muscle and the *mdx* and Sgcd-null mouse models of muscular dystrophy, making possible the first comprehensive, comparative proteomic study of the DGC in skeletal muscle in mice.

This MS analysis of the DGC in skeletal muscle was successful with respect to several bioanalytical achievements. First, the nonionic detergent digitonin effectively promoted the water solubilization of membrane-bound DGC, facilitating downstream biochemical analyses. Second, lectin-affinity chromatography made it possible to enrich for detergent-solubilized glycosylated proteins. Third, sucrose-density gradient centrifugation proved an effective method for sizing glycoprotein complexes. Fourth, the FASP method facilitated removal of MS-incompatible analytes (*i.e.* sucrose, detergent, salts) from sucrose-gradient protein fractions prior to dMS (56). Fifth, the dMS acquisition scheme enabled us to acquire higher quality MS/MS spectra of targeted peptide ions present in the tryptic-peptide samples from the complex. As such, our proteomic workflow facilitated the in-depth MS-based analysis of the DGC in mouse skeletal muscle tissue.

A technical strength of our label-free proteomic workflow was the use of trypsin-digested, iodoacetic-acid labeled bovine serum albumin (BSA) as an internal standard to normalize measurements across samples from WT, *mdx*, and Sgcd-null skeletal muscle. Previous label-free proteomic studies used trypsin-digested iodoacetamide-labeled BSA for this purpose (57) (58). However, a recent proteomic study in *Arabidopsis thaliana* showed that the intensities of BSA protein ions were sufficiently reproducible for protein normalization (58). Our proteomic study builds upon this theme, demonstrating that iodoacetic-acid labeled BSA is a superior to iodoacetamide labeled BSA as an internal standard, based upon the fact that the majority of proteomic samples are alkylated with iodoacetamide. This ready distinction is especially important because of the high degree of conservation of serum albumins across vertebrate species (*i.e.* >70% protein identity between human, monkey, dog, cow, mouse, and rat) (59) The iodoacetic-acid labeled cysteine-containing BSA peptides harbor a +1 AMU mass shift difference (+58) relative to sample-derived cysteine containing BSA peptides labeled with iodoacetamide (+57). On a high-resolution mass spectrometer, this mass differential is sufficient to distinguish sample-derived BSA peptides from standard BSA peptides.

Another advantage of using iodoacetic-acid labeled BSA as an internal standard is the fact that trypsin-digested iodoacetic-acid BSA contains 26 tryptic cysteine-containing peptides that can be used to normalize quantified proteins across samples. Theoretically, these 26 iodoacetic-acid labeled cysteine peptides (restricted to 2+ and 3+ charged ions) constitute a library that provides ∼31% proteomic coverage of the BSA standard, based upon their ionization in the 400–1250 *m*/*z* MS1 window during a dMS experiment. In essence they function as external standards for the detection of systematic error that may arise during sample handling (*i.e.* sample peptide fraction-SCX spin column, sample processing-C18 spin column), or changes in sensitivity of the mass spectrometer that may arise during the acquisition of peptide ions in MS1 and dMS experiments (*e.g.* because of ion suppression, problems with the nano-LC system). Obviously the introduction of protein/peptide standards into an experimental sample is not without limitations; an excess of standard can induce ion suppression and reduce the amount of proteins detected in the experimental sample. Empirical studies in our laboratory have shown that a 1:75 molar ratio of standard peptide to sample peptide had no deleterious impact on the identification and quantification of proteins in complex peptide samples (unpublished data). An optimal molar ratio of BSA to target protein (1–4 pmol to 5–25 μg of protein) was also obtained in *Arabidopsis thaliana* (58).

A final advantage of our targeted proteomic workflow is that selective-reaction monitoring (SRM) methods combined with stable-isotope dilution (SID) can be easily incorporated into our label-free proteomic workflow to quantify the DGC, and also to measure dynamic changes in the DGC under a variety of experimental conditions (60–62). We anticipate that it will be possible to adapt our label-free proteomic workflow for the study of changes in the composition of other plasma membrane-related protein complexes that are linked to disease in tissues other than skeletal muscle.

##### Validation of DGC Composition and Potential Identification of Novel DGC Components

Our discovery by proteomic analysis of sucrose-gradient fractions that DGC subunits resolved into a single cluster (K5) was validated using a coimmunoprecipitation study and corroborates earlier findings suggesting that the DGC is a stable plasma-membrane protein complex (reviewed in (63)). Our analysis revealed comigration between the known DGC components and a subset of proteins that had not previously been implicated in the DGC in WT mice, and reductions in these associations in mouse models of muscular dystrophy. Therefore, these proteins are candidates as novel DGC-interacting proteins. As protein-protein interaction maps are built from published data based either on actual experiments or literature-based methodologies, novel protein-protein interactions cannot be determined. However, our analysis provides a powerful enrichment strategy to provide a set of candidates which need to be tested for physical binding to the DGC in future experiments. This includes the application of stable-isotope dilution mass spectrometry (SID-MS) methods to validate and monitor the DGC across different muscular dystrophy models.

##### Putative Compensatory Cell Adhesion Pathways in Muscular Dystrophy Models

A major finding from our study was the detection of cluster-specific protein complexes in the skeletal muscle of the *mdx* and Sgcd-null models of muscular dystrophy. Our direct comparison of such complexes between WT and mutant mice allowed us to establish if protein complexes were up- or downregulated in the latter by combining the information present in Fig. 4 and supplemental Fig. S3. Interestingly, there were similarities and differences in clustered protein signals between the mdx and Sgcd mice (Fig. 4*A*). Inspection of clusters K6 (mdx), K9 (mdx), K12 (mdx), and K13 (Sgcd) demonstrate an overlap in inflammatory and cell adhesion protein network processes. Specific proteins representative of these broad protein network processes might reflect differences in the pathological state of each between the mdx and Sgcd muscular dystrophies. For example, asynchronously regenerating microenvironments have been identified as an underlying driver of fibrosis and failed regeneration in muscular dystrophies (28). Differences in clustered protein networks between the mdx and Sgcd mice might reflect dissimilarities in asynchronously regeneration between the muscular dystrophy models. Future experimentation should be able to confirm this hypothesis between mdx and Sgcd mice. Furthermore, our bioinformatic analysis showed that the NFκB pathway was significantly up-regulated in both mouse models for muscular dystrophy, and that the majority of the NFκB regulated proteins were present in three clusters: K2, K10, and K11. The fact that cluster K11 exhibited the most PPIs suggests that these proteins form an NFκB-regulated protein complex. Our further bioinformatic analysis of the NFκB-regulated complex showed that it was enriched for several integrins, among which some contribute to the development of muscle tissue (ITGA5 and ITGA6) and others link the extracellular matrix to the plasma membrane (ITGA7). Indeed, recent studies showed that several integrins (*i.e.* Itga2, Itga5, Itgb1, and Itgb4) are NFκB regulated (64–69). These findings suggest that integrin-based cellular function (*i.e.* cell adhesion) is important for normal muscle development and activity, and that NFκB-regulated pathways may become activated when muscle homeostasis is disrupted.

Interestingly, the only pharmacological intervention for Duchenne Muscular Dystrophy (DMD) is treatment with glucocorticoids, agents that are thought to act by suppressing inflammation via inhibition of NFκB, stabilizing the sarcolemma, reducing necrosis, and increasing muscle mass (70). Thus, glucocorticoid therapy slows the progression of weakness, reduces the development of scoliosis, and delays respiratory insufficiency (71). However, long-term glucocorticoid treatment in DMD patients can result in detrimental side effects such as the development of Cushingoid syndrome, weight gain, compromised bone health, and dose-dependent growth retardation (71). Moreover, in *mdx* mice long-term glucocorticoid application promotes muscle wasting (72). Therefore, despite the benefits of glucocorticoids in treating DMD patients, potentially deleterious effects in the long term might limit their therapeutic value.

Recently, a promising novel drug for the treatment of DMD, VBP15, was discovered. Like glucocorticoids, it has beneficial NFκB-inhibiting effects, but it does not transactivate the glucocorticoid receptor (GR) and thus does not reproduce the deleterious side effects of steroids with respect to muscle repair and strength (50). The beneficial effects of VBP15 may result from altered regulation of specific genes, because the transcriptional activities of the glucocorticoid receptor and NFκB have been described to physically interact (73). We speculate that although glucocorticoids reduce muscle necrosis, their immunosuppressive effects interfere with muscle regeneration, consistent with the fact that analyses of glucocorticoid-treated *mdx* mice provided no evidence for increased regeneration (70). In contrast, drugs (like VBP15) that lack immunosuppressive effects could inhibit specifically NFκB-mediated transcription. Moreover, despite the fact that NFκB signaling normally promotes cell adhesion, the apparent compensatory up-regulation of various integrin signaling pathways in the skeletal muscle of *mdx* and Sgcd-null skeletal mice may be sufficient to rescue muscle regeneration in these contexts. Quantitative protein profiling of the integrin networks expressed in *mdx* and Sgcd-null mice in the context of glucocorticoid and VBP15 treatment is expected to address these possibilities in the future.

This study describes an analytical workflow to study the composition of plasma membrane protein complexes in skeletal muscle tissue. Under the experimental conditions employed in this study our proteomic workflow is only amenable to the study of the DGC expressed in mouse skeletal muscle. It remains to be determined whether our biochemical workflow is suitable for to the study of other well-characterized membrane protein complexes in mammalian systems. More importantly, our analytical framework validated components of the DGC in skeletal muscle tissue and showed how mutations in the DGC perturbed the composition of the DGC in established mouse models of muscular dystrophy. The molecular interrogation of the DGC in skeletal muscle tissue will facilitate the identification of novel components of the DGC, provide deeper molecular insights into how drugs influence the biochemical composition of the DGC, and uncover active compensatory pathways and/or mechanisms at the molecular and cellular level to influence the skeletal muscle phenotype in muscle dystrophies. Finally, the proteomic findings will be of general interest to skeletal muscle biologists, as many of the reported proteins may encode novel biomarkers of skeletal muscle diseases.

## Supplementary Material

Supplemental Data

## Footnotes

^1^ The abbreviations used are:

DG: Dystroglycan
DGC: Dystrophin-glycoprotein complex
DMD: Dystrophin
α-DG: Dystroglycan alpha subunit
β-DG: Dystroglycan beta subunit
DTNA: alpha-dystrobrevin
SNTA1: alpha-syntrophin-1
SNTB1: beta-syntrophin-1
NNOS: neuronal nitric oxide synthase
SGCA: alpha-sarcoglycan
SGCB: beta-sarcoglycan
SGCD: delta-sarcoglycan
SGCG: gamma-sarcoglycan
SSPN: Sarcospan
2DGE: 2-dimensional gel electrophoresis
dMS: Directed mass spectrometry
FASP: Filter-aided sample preparation
SCX: Strong-cation exchange
LC: Liquid chromatography
BSA: Bovine serum albumin
AMU: Atomic mass unit
MW: Molecular weight
FDR: False discovery rate
Gene ontology: (GO)
Wild type: (WT)
Protein-protein interaction: (PPI)
Plectin-1: (PLEC1)
DES: Desmin
UTRN: Utrophin
GRB2: Growth factor receptor-bound 2
PLCB2: Phospholipase C-beta-2
NMJ: Neuromuscular junction
NFκB: Nuclear factor kappa Beta
ITGA7: Integrin-alpha-7
ITGB1: Integrin-beta-1
ITGA5: Integrin-alpha-5
ITGA6: Integrin-alpha-6
ITGAM: Integrin-alpha-M
ITGB2: Integrin-beta-2
ITGB3: Integrin-beta-3
sCK: Serum creatine kinase
B2M: Beta-alpha-2-microglobulin
CLU: Clusterin
EGFR: Epidermal growth factor receptor
FN1: Fibronectin
TTN: Titin
SRM: Selective-reaction monitoring
SID: Stable-isotope dilution
GR: Glucocorticoid receptor
WGA: Wheat-germ agglutinin
SDS-PAGE: Sodium dodecyl sulfate-polyacrylamide gel electrophoresis
DTT: Dithiothreitol
ACN: Acetonitrile
Q-TOF: Quadrupole Time-of-Flight.
