# Supplementary material for: Molecular Signatures of Membrane Protein Complexes Underlying Muscular Dystrophy
Source: Mol Cell Proteomics. 2016 Apr 20;15(6):2169–85. doi: 10.1074/mcp.M116.059188 (PMC5083101; doi:10.1074/mcp.M116.059188)
Supplement: Supplemental Data [file supp_15_6_2169__index.html]

Molecular signatures of membrane protein complexes underlying muscular dystrophy — Molecular Signatures of Membrane Protein Complexes Underlying Muscular Dystrophy — Molecular Signatures of Membrane Protein Complexes — Supplemental Data 

# Molecular Signatures of Membrane Protein Complexes Underlying Muscular Dystrophy

## Supplemental Data

- Supplemental Figure 1 (.pdf, 415 KB) - Sucrose Gradient
- Supplemental Figure 2 (.jpg, 918 KB) - Silver stained gel
- Supplemental Figure 3 (.pdf, 2.4 MB) - Cluster analysis
- Supplemental Figure 4 (.pdf, 3.7 MB) - Expression Networks
- Supplemental Figure 5 (.pdf, 897 KB) - DGC network
- Supplemental Table 1 (.xlsx, 28 KB) - BSA peptides
- Supplemental Table 2 (.xlsx, 10 KB) - Directed MS versus DDA of BSA peptides
- Supplemental Table 3 (.xlsx, 14 KB) - K-means clusters
- Supplemental Table 4 (.xlsx, 16 KB) - Protein Networks
- Supplemental Table 5 (.xlsx, 161 KB) - Mass spectrometry metrics for protein identifications
- Supplemental Table 6 (.xlsx, 7 KB) - K-means clusters of integrins
- Supplemental Table 7 (.xlsx, 4.2 MB) - Peptide sequence identifications
- Supplemental Figure and Table Legends (.pdf, 12 KB) - Supplemental Figure and Table Legends
