# Supplementary figures and images for: Molecular Signatures of Membrane Protein Complexes Underlying Muscular Dystrophy
Source: Mol Cell Proteomics. 2016 Apr 20;15(6):2169–85. doi: 10.1074/mcp.M116.059188 (PMC5083101; doi:10.1074/mcp.M116.059188)

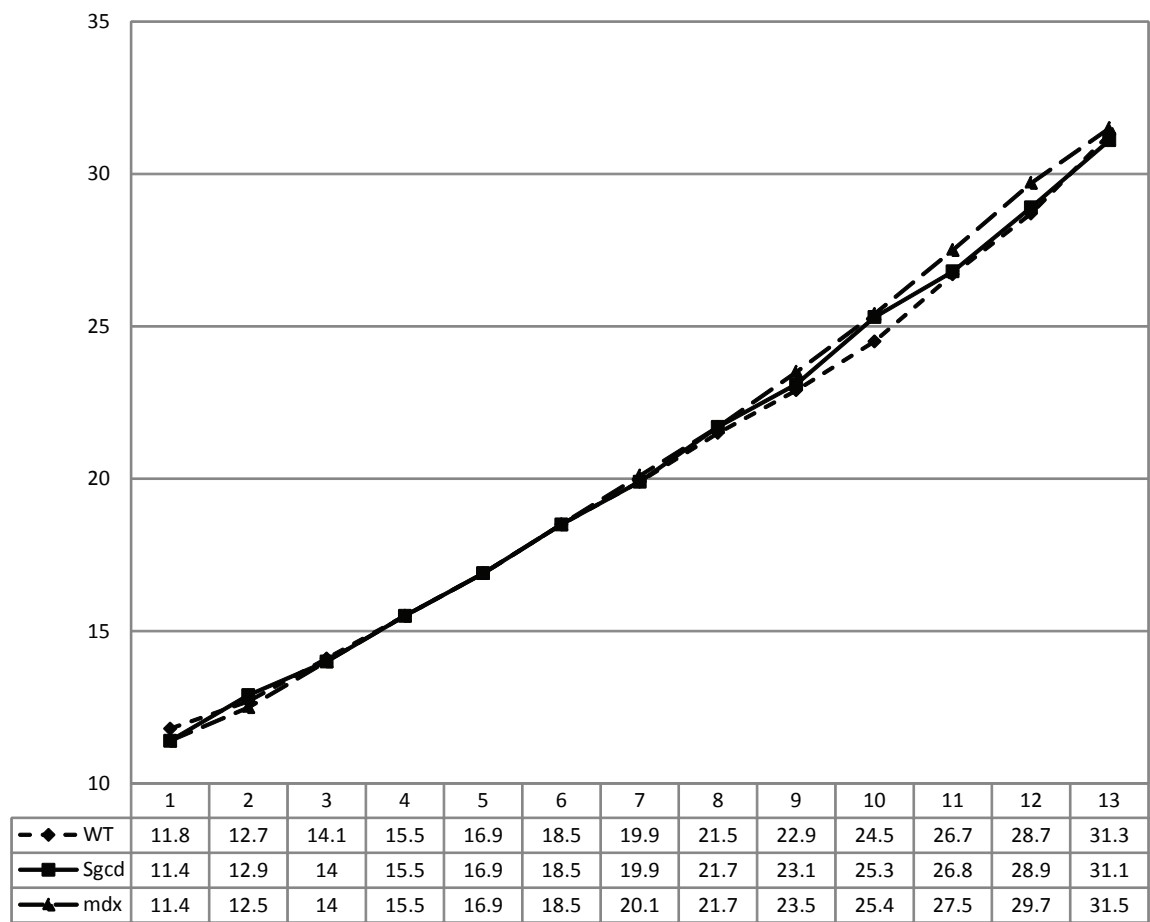

Supplemental Figure 1

Supplement: Supplemental Data [file 10.1074_M116.059188_mcp.M116.059188-1.pdf]

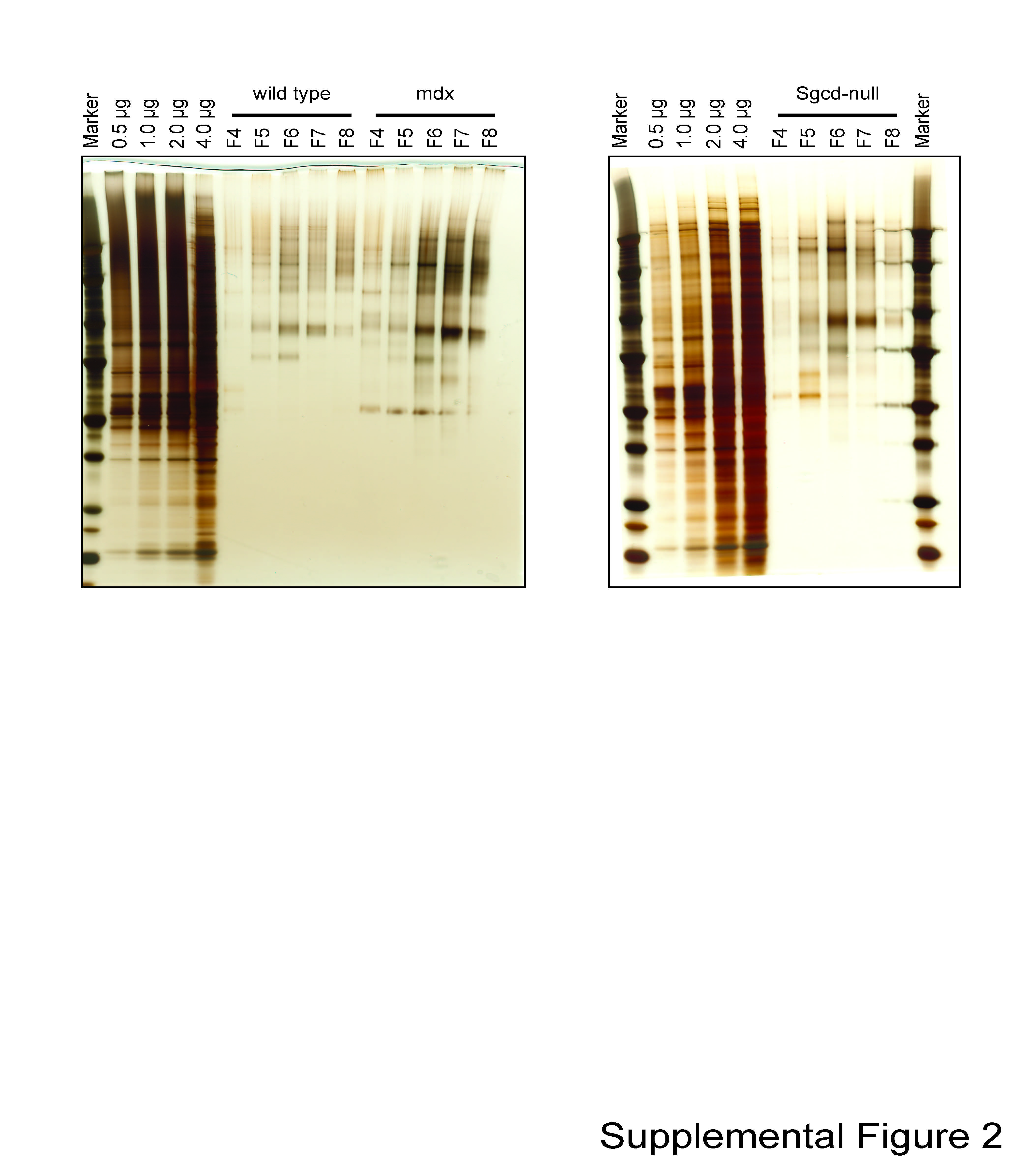

Supplement: Supplemental Data [file 10.1074_M116.059188_mcp.M116.059188-2.jpg]

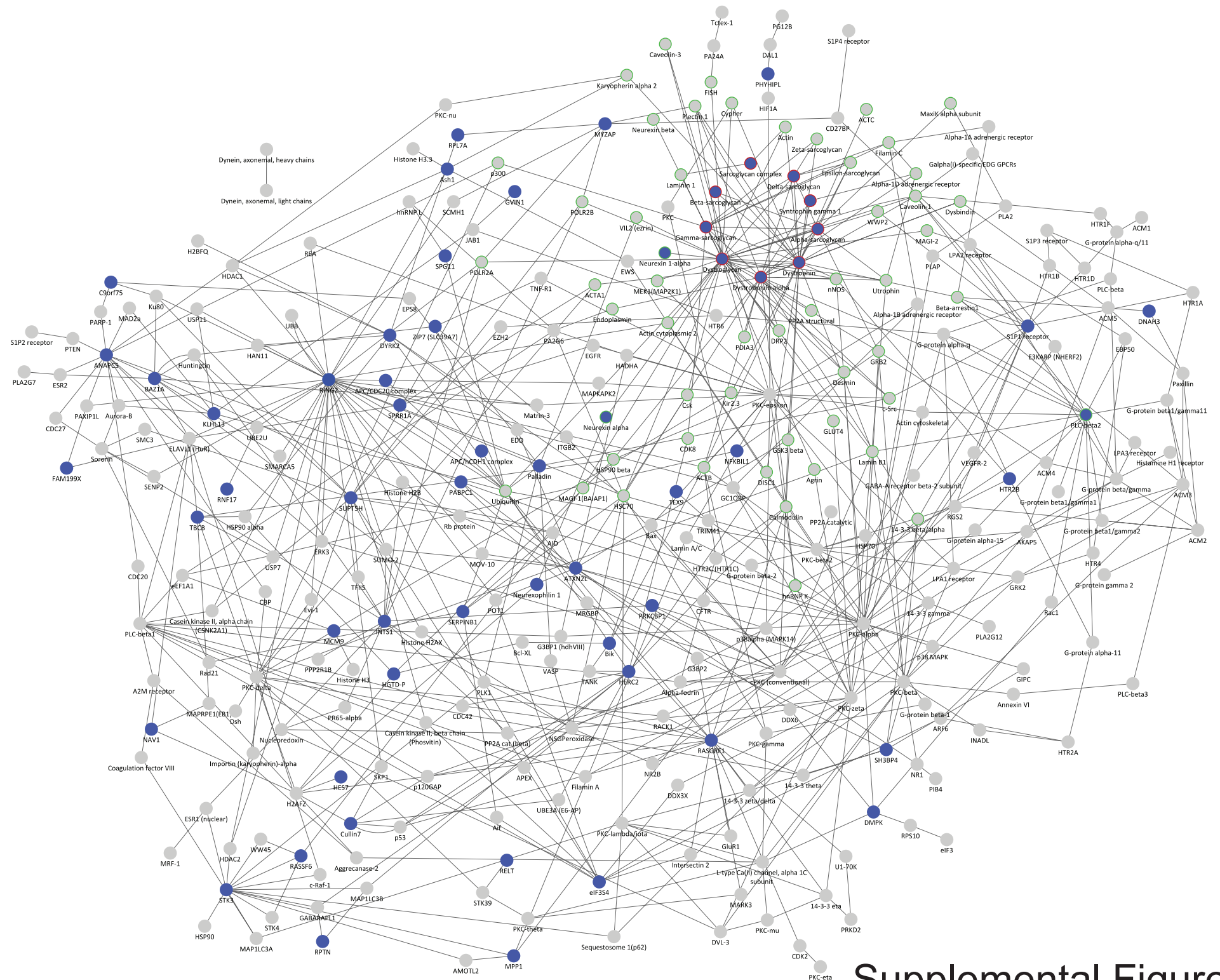

Supplement: Supplemental Data [file 10.1074_M116.059188_mcp.M116.059188-4.pdf]

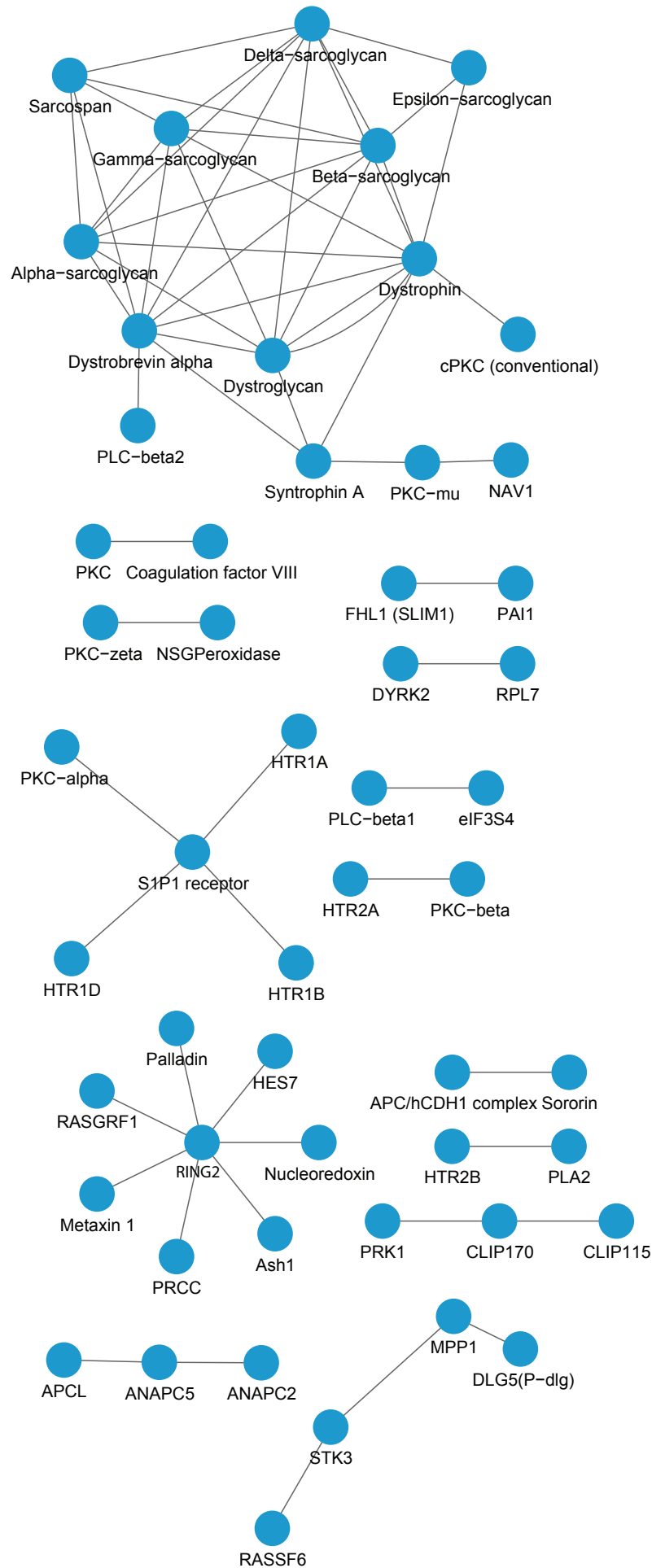

Supplement: Supplemental Data [file 10.1074_M116.059188_mcp.M116.059188-5.pdf]
