# Supplementary material for: Molecular Signatures of Membrane Protein Complexes Underlying Muscular Dystrophy
Source: Mol Cell Proteomics. 2016 Apr 20;15(6):2169–85. doi: 10.1074/mcp.M116.059188 (PMC5083101; doi:10.1074/mcp.M116.059188)
Supplement: Supplemental Data [file 10.1074_M116.059188_mcp.M116.059188-3.pdf]

Cluster K1

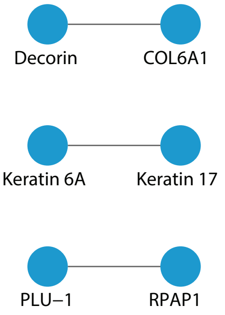

Cluster K2

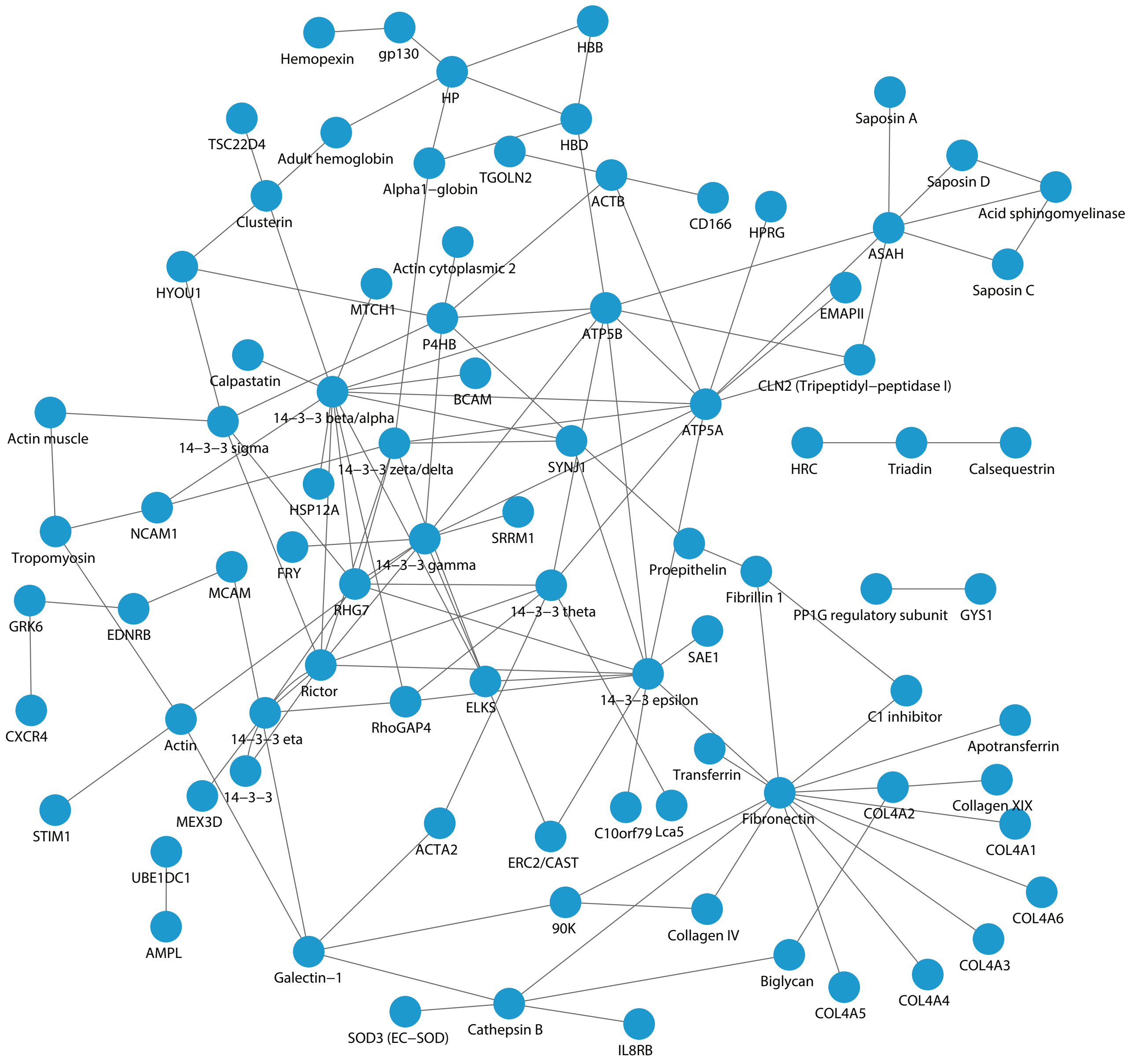

Supplemental Figure 3A

Cluster K3

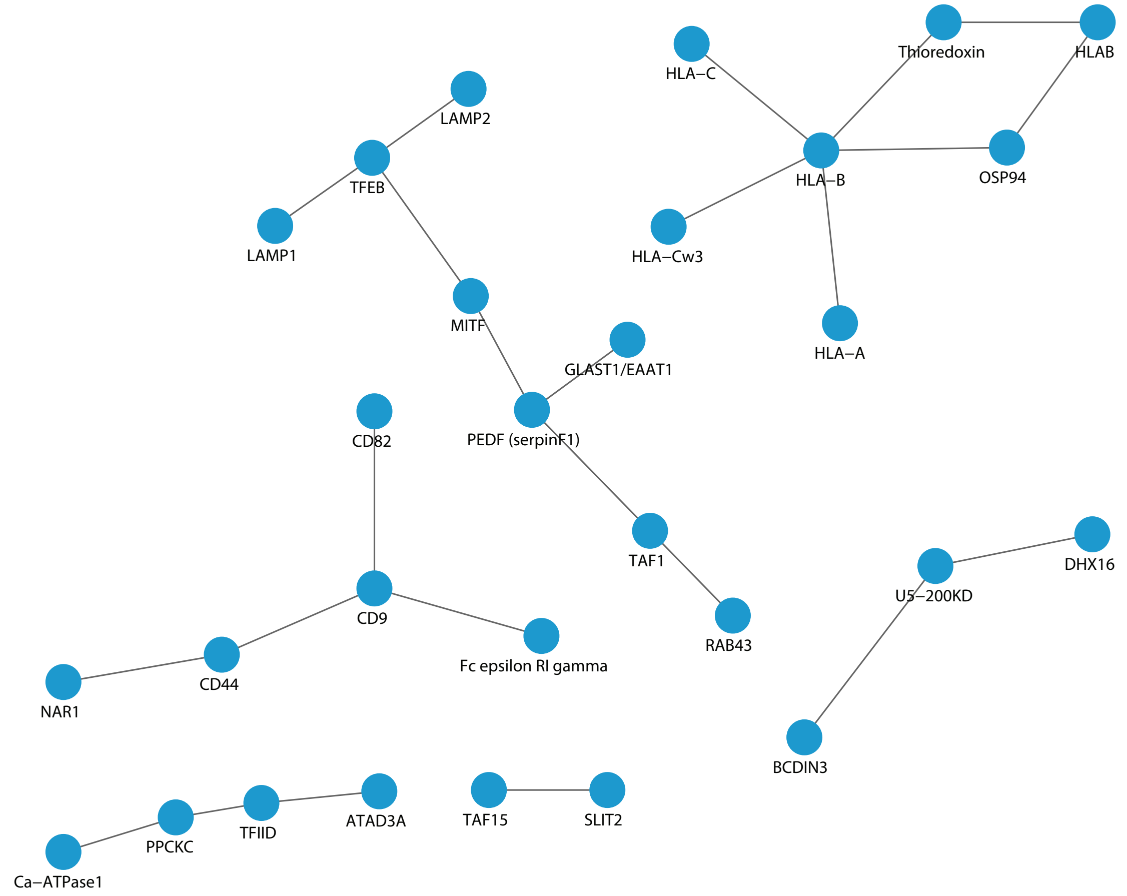

Cluster K4

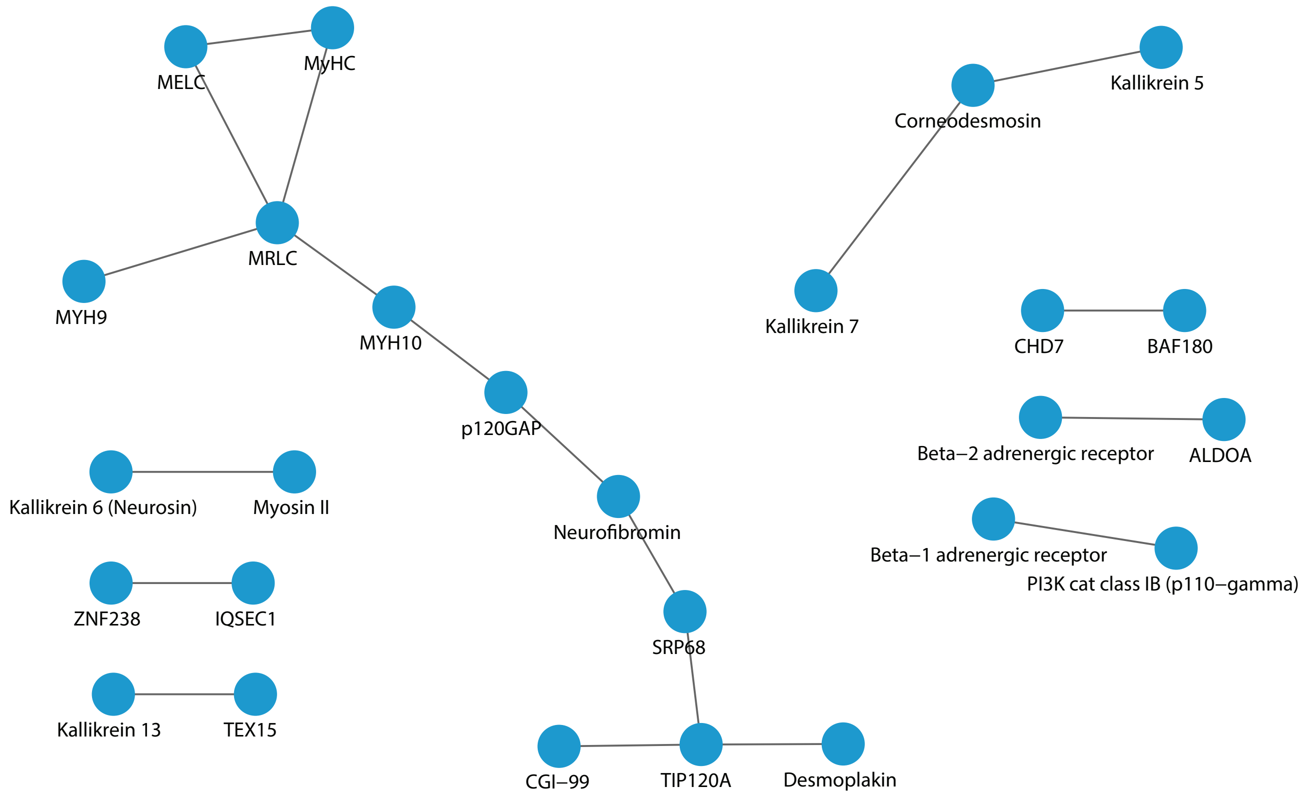

Supplemental Figure 3B

Cluster K5

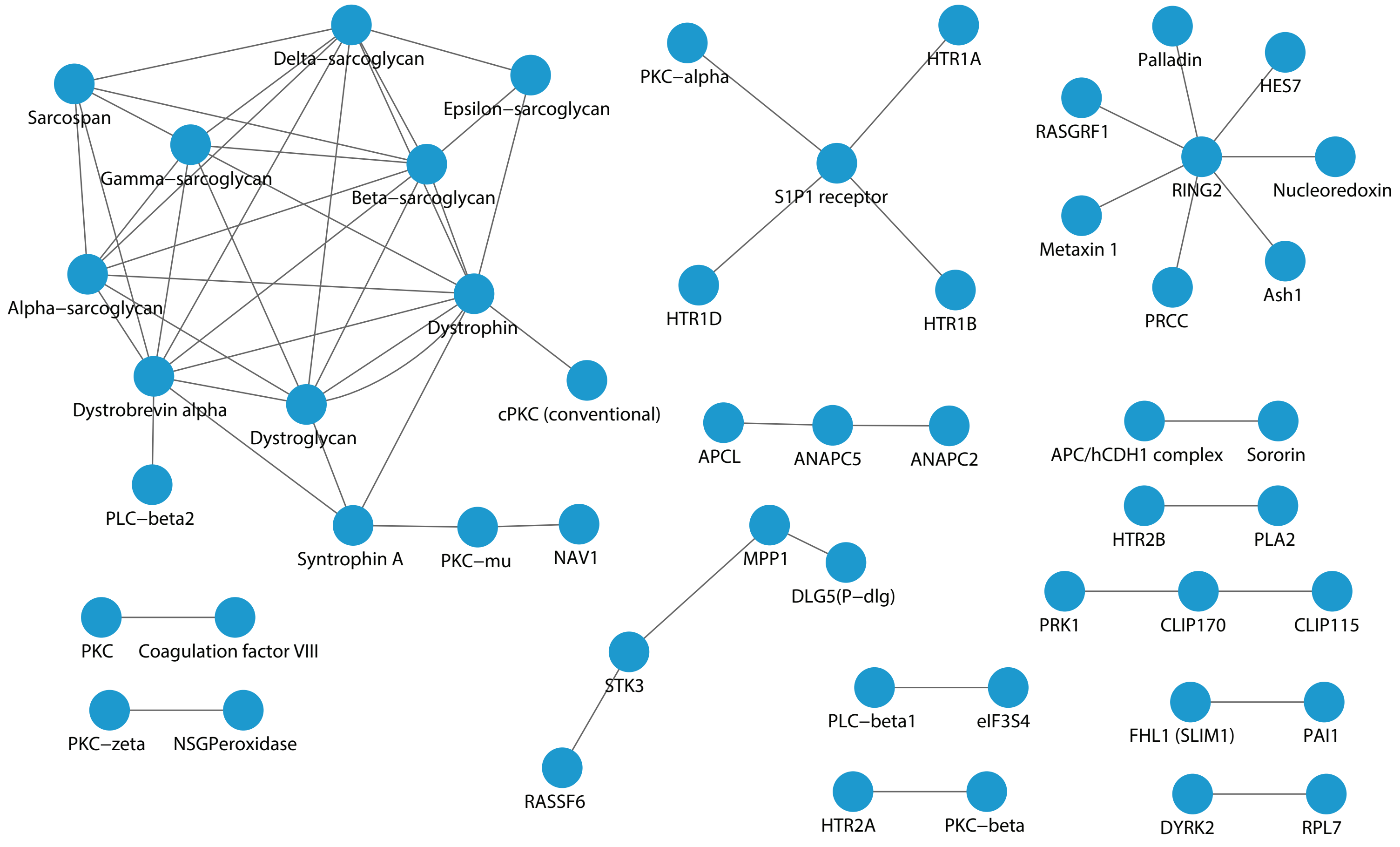

Cluster K6

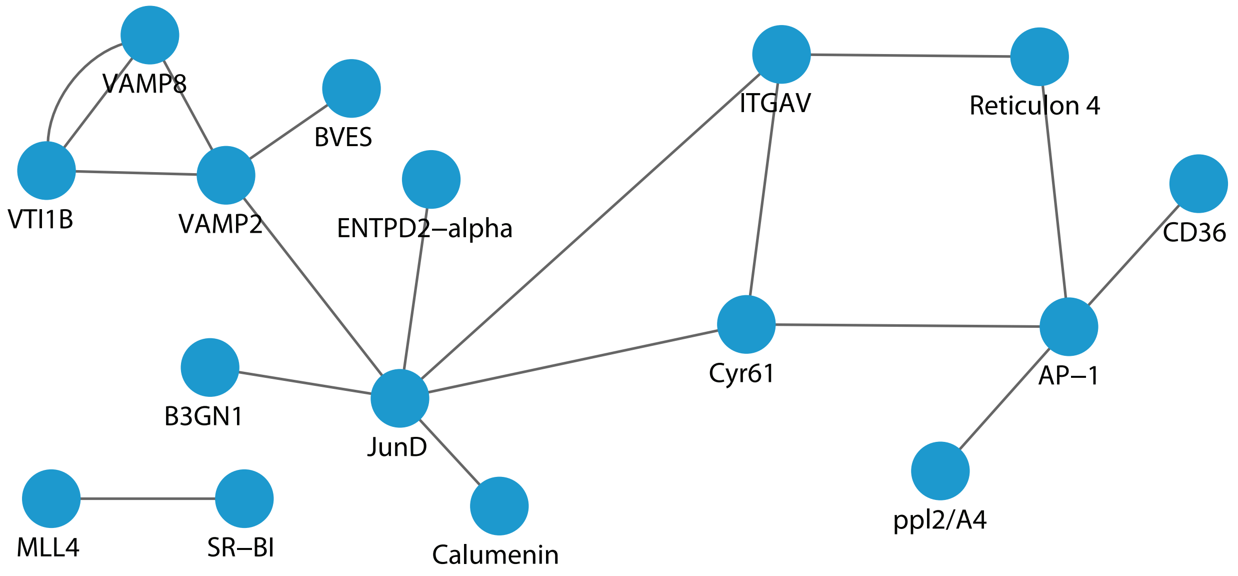

Supplemental Figure 3C

Cluster K7

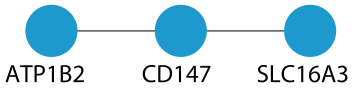

Cluster K8

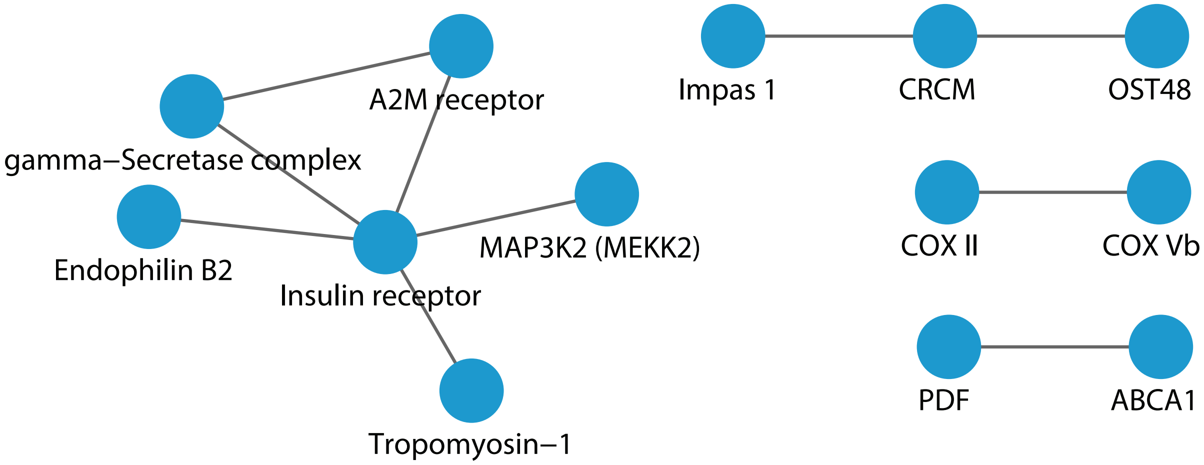

Cluster K9

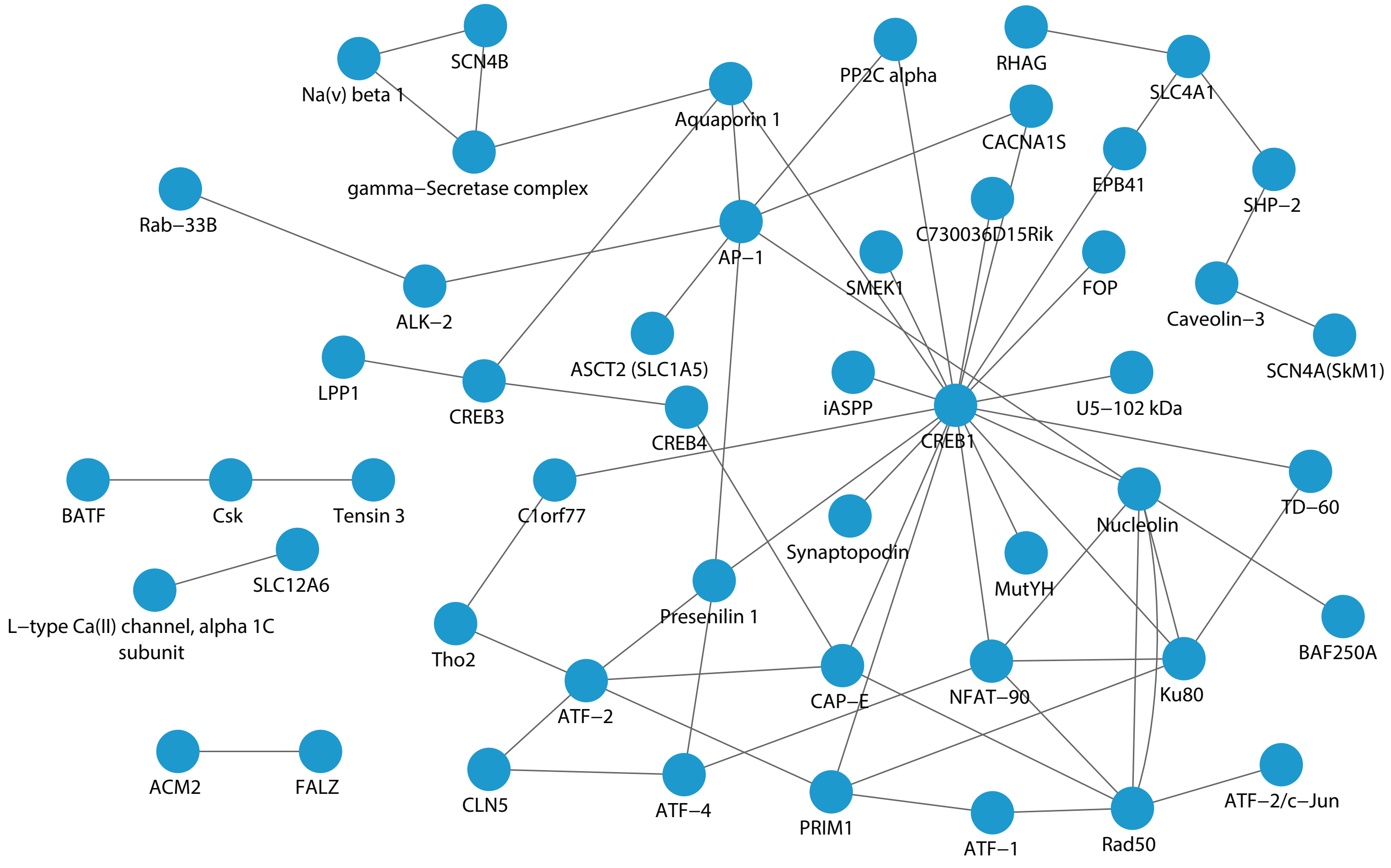

Supplemental Figure 3D

Cluster K10

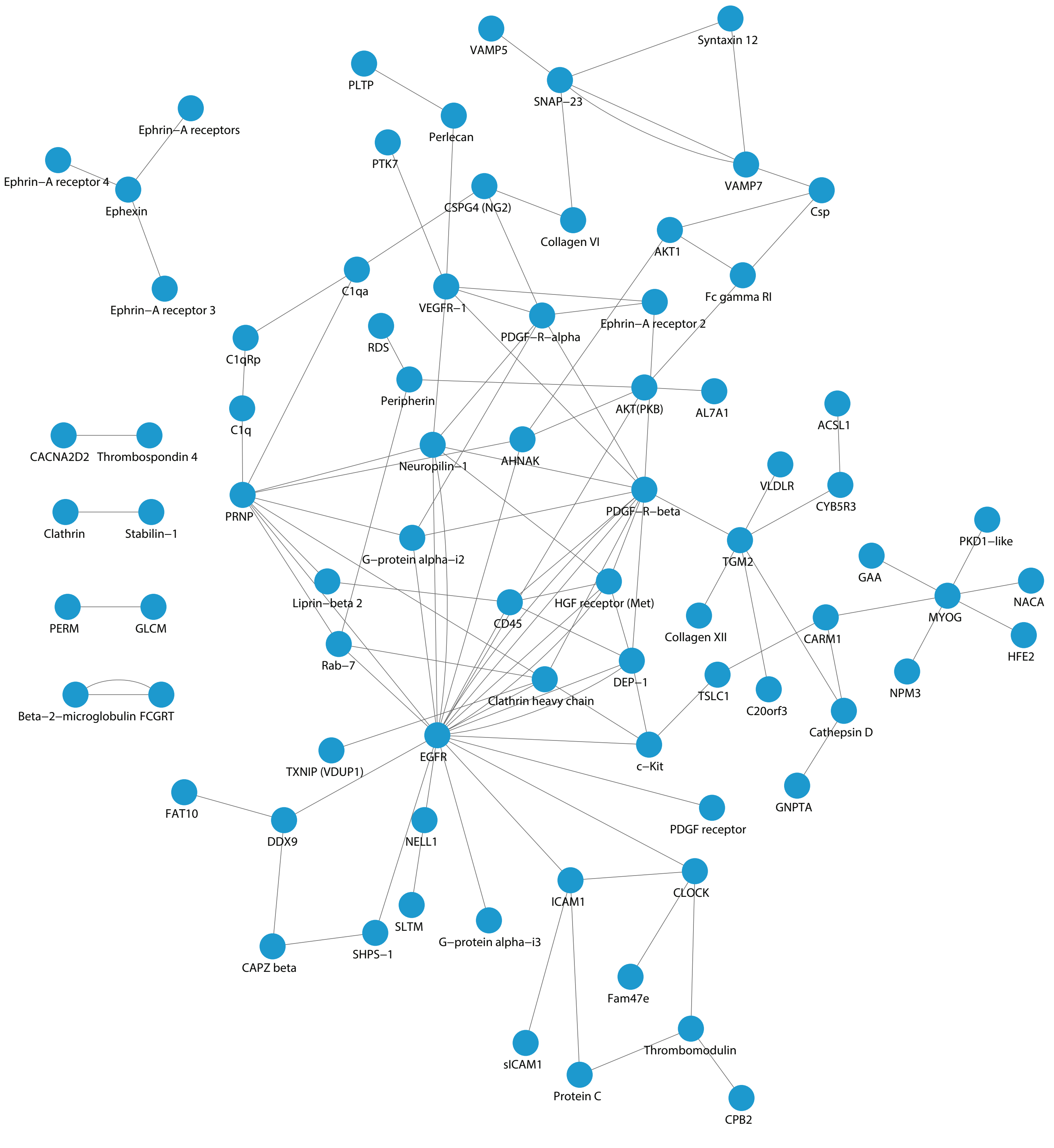

Supplemental Figure 3E

Cluster K11

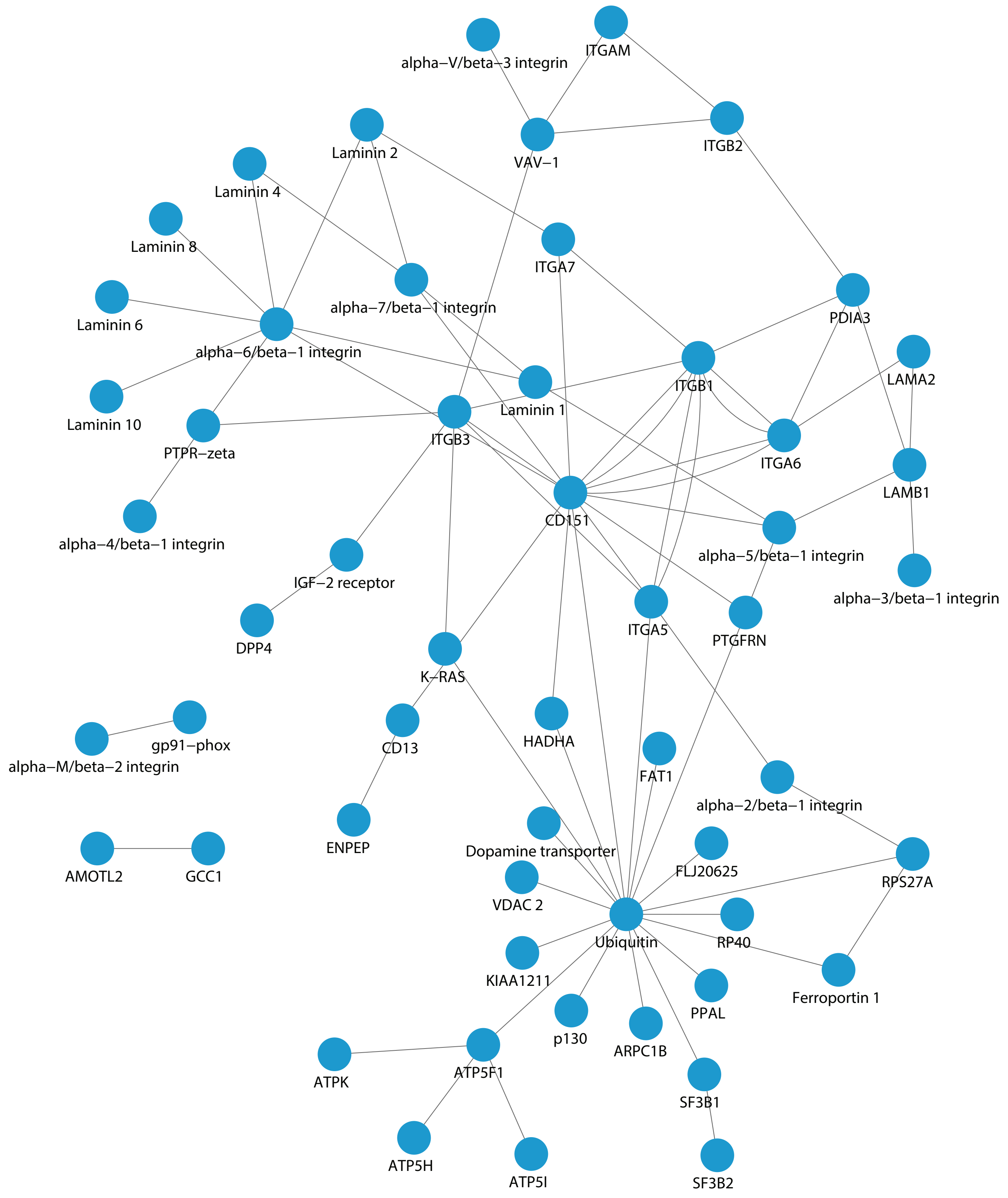

Supplemental Figure 3F

Cluster K12

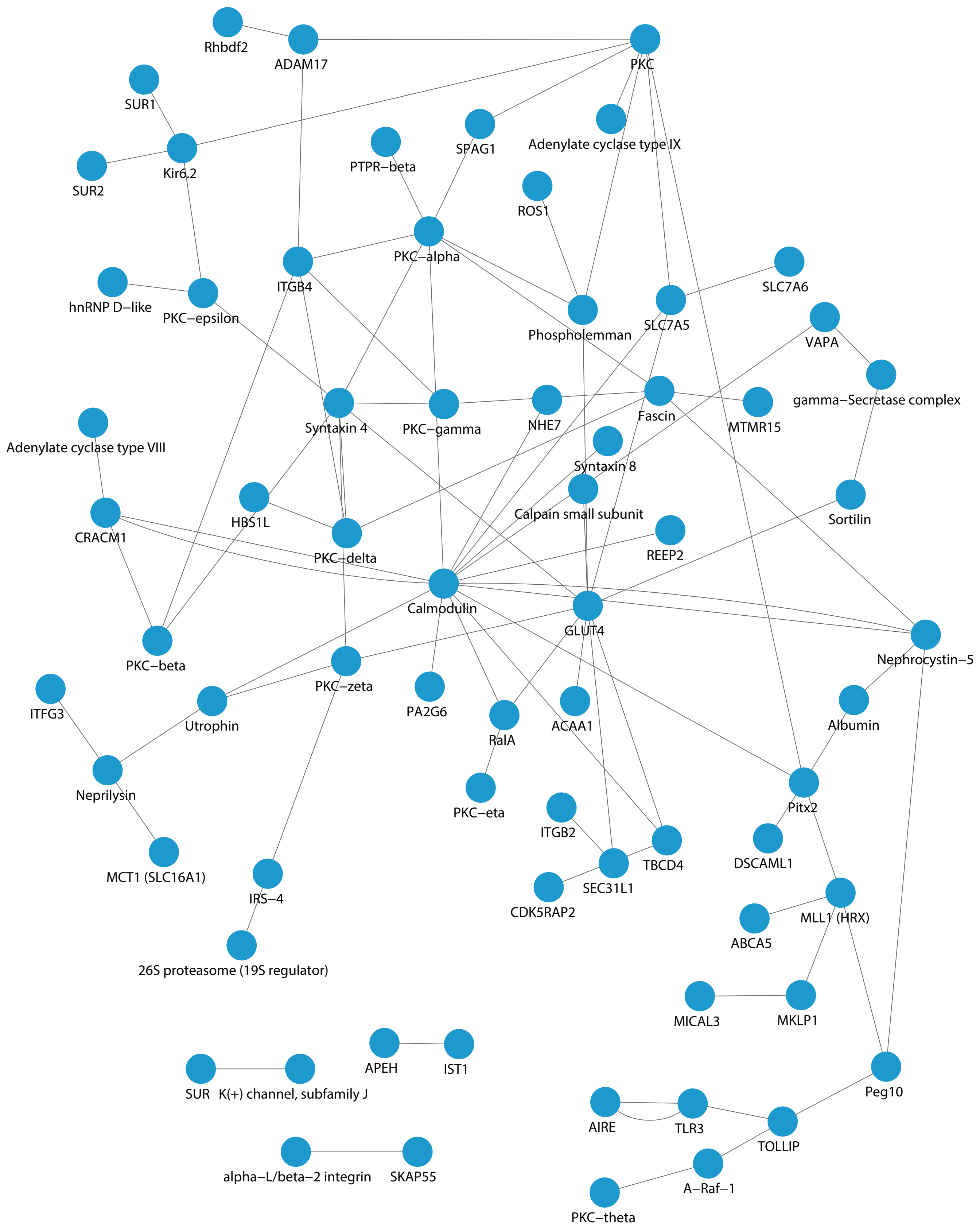

Supplemental Figure 3G

Cluster K13

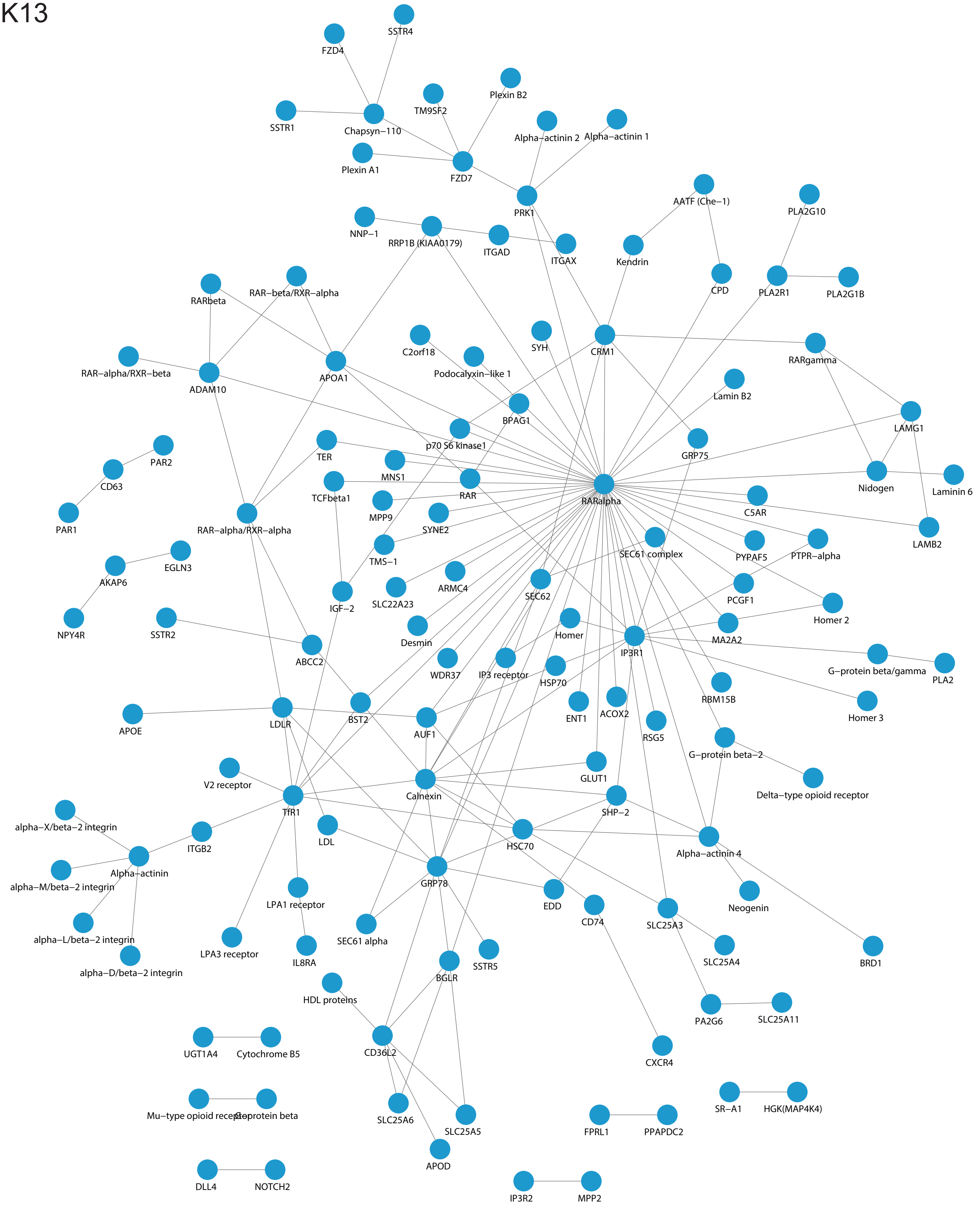

Cluster K14

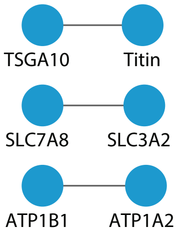

Supplemental Figure 3H
