# Supplementary material for: Molecular Signatures of Membrane Protein Complexes Underlying Muscular Dystrophy
Source: Mol Cell Proteomics. 2016 Apr 20;15(6):2169–85. doi: 10.1074/mcp.M116.059188 (PMC5083101; doi:10.1074/mcp.M116.059188)
Supplement: Supplemental Data [file 10.1074_M116.059188_mcp.M116.059188-13.pdf]

Supplemental Figure/Table Legends

Supplemental Figure 1: Sucrose-gradient fractionation. Graph depiction of the sucrose percentage in each fraction that demonstrated comparable gradients between mouse models.

Supplemental Figure 2: Silver staining of sucrose-gradient fractions. Protein distribution and abundance prior to further fractionation by ion-exchange chromatography are shown.

Supplemental Figure 3: PPI maps for each *K*-means cluster. Protein-protein interactions based on direct binding were generated for each cluster, using the *GeneGo* software.

Supplemental Figure 4: Protein-protein interaction (PPI) map of DGC-associated proteins. Proteins present in cluster K5 are shown in blue, and among these the DGC-related proteins have a red border; others are shown in gray. Proteins directly associated with the DGC (first neighbors) have a green border.

Supplemental Figure 5: Protein-protein interactions of DGC-associated proteins. Protein-protein interactions present in cluster K5 are shown.

Supplemental Table 1: Total intensities of 14 iodoacetic-labeled BSA peptides used for protein sample normalization.

Supplemental Table 2: dMS protocol vs. DDA protocol.

Supplemental Table 3: Process network analysis of K-means clusters. Statistically significant process networks were determined for each cluster, using the *GeneGo* software. In each case the ratio represents the number of proteins in the specified cluster over the total number of proteins in the network

Supplemental Table 4: Transcriptional network analysis on proteins whose expression was elevated at least 2-fold in the Sgcd-null or *mdx* vs WT samples.

Supplemental Table 5: List of identified proteins across the WT, Sgcd-null and *mdx* samples.

Supplemental Table 6: List of integrins detected in WT, Sgcd-null and *mdx* samples.

Supplemental Table 7: List of all peptide sequence identifications and post-translational modifications.
